# Prognostic Ferroptosis-Related lncRNA Signatures Associated With Immunotherapy and Chemotherapy Responses in Patients With Stomach Cancer

**DOI:** 10.3389/fgene.2021.798612

**Published:** 2022-01-03

**Authors:** Donglin Lai, Lin Tan, Xiaojia Zuo, DingSheng Liu, Deyi Jiao, Guoqing Wan, Changlian Lu, Dongjie Shen, Xuefeng Gu

**Affiliations:** ^1^ Shanghai Key Laboratory of Molecular Imaging, Zhoupu Hospital, Shanghai University of Medicine and Health Sciences, Shanghai, China; ^2^ School of Health Science and Engineering, University of Shanghai for Science and Technology, Shanghai, China; ^3^ The Affiliated Zhuzhou Hospital Xiangya Medical College CSU, Zhuzhou, China; ^4^ School of Pharmacy, Shanghai University of Medicine and Health Sciences, Shanghai, China; ^5^ Department of General Surgery, Ruijin Hospital Luwan Branch, Shanghai Jiaotong University School of Medicine, Shanghai, China

**Keywords:** stomach cancer, ferroptosis, prognostic, lncRNA, immunotherapy, chemotherapy

## Abstract

Ferroptosis is associated with the prognosis and therapeutic responses of patients with various cancers. LncRNAs are reported to exhibit antitumor or oncogenic functions. Currently, few studies have assessed the combined effects of ferroptosis and lncRNAs on the prognosis and therapy of stomach cancer. In this study, transcriptomic and clinical data were downloaded from TCGA database, and ferroptosis-related genes were obtained from the FerrDb database. Through correlation analysis, Cox analysis, and the Lasso algorithm, 10 prognostic ferroptosis-related lncRNAs (*AC009299.2*, *AC012020.1*, *AC092723.2*, *AC093642.1*, *AC243829.4*, *AL121748.1*, *FLNB-AS1*, *LINC01614*, *LINC02485*, *LINC02728*) were screened to construct a prognostic model, which was verified in two test cohorts. Risk scores for patients with stomach cancer were calculated, and patients were divided into two risk groups. The low-risk group, based on the median value, had a longer overall survival time in the KM curve, and a lower proportion of dead patients in the survival distribution curve. Potential mechanisms and possible functions were revealed using GSEA and the ceRNA network. By integrating clinical information, the association between lncRNAs and clinical features was analyzed and several features affecting prognosis were identified. Then, a nomogram was developed to predict survival rates, and its good predictive performance was indicated by a relatively high C-index (0.67118161) and a good match in calibration curves. Next, the association between these lncRNAs and therapy was explored. Patients in the low-risk group had an immune-activating environment, higher immune scores, higher TMB, lower TIDE scores, and higher expression of immune checkpoints, suggesting they might receive a greater benefit from immune checkpoint inhibitor therapy. In addition, a significant difference in the sensitivity to mitomycin. C, cisplatin, and docetaxel, but not etoposide and paclitaxel, was observed. In summary, this model had guiding significance for prognosis and personalized therapy. It helped screen patients with stomach cancer who might benefit from immunotherapy and guided the selection of personalized chemotherapeutic drugs.

## Introduction

Stomach cancer is a malignant tumor worldwide. Research has shown that gastric cancer ranks fifth in global incidence and sixth in mortality, with over one million new cases and an estimated 769,000 deaths in 2020 (Sung et al., 2021). The AJCC/UICC TNM staging system has been used to predict prognosis for many years (Marano et al., 2015). However, due to the complexity and high heterogeneity of stomach cancer, patients with the same TNM stratification sometimes present distinct prognoses. Hence the development of novel and effective biological markers to predict prognosis is urgently needed. Treatment strategies for stomach cancer currently include endoscopic resection, surgery, perioperative or adjuvant chemotherapy, and targeted therapy (Smyth et al., 2020). The treatment efficacy in patients is affected by the chosen treatment strategy. Hence, biological markers of gastric cancer for personalized treatment, including selecting effective strategies and avoiding excessive treatment, are important.

Ferroptosis is a form of cell death that differs from apoptosis and autophagy in morphology, biochemistry, and genetics (Dixon et al., 2012). Ferroptosis-inducing factors result in the accumulation of reactive oxygen species by affecting glutathione peroxidase activities, which regulates cell death (Li et al., 2020). Recently, the involvement of ferroptosis in tumor suppression has received increasing attention (Lang et al., 2019; Liang et al., 2019; Lei et al., 2020; Tang et al., 2020). In addition, ferroptosis is associated with cancer therapy responses, such as immunotherapy, radiotherapy, and chemotherapy. CD8^+^ T cells activated by immunotherapy enhance the ferroptosis-specific lipid peroxidation of tumor cells to improve the antitumor efficacy of immunotherapy (Wang W. et al., 2019). Biomimetic magnetic nanoparticles, Fe_3_O_4_-SAS@PLT, sensitize cells to ferroptosis, improving the cancer immunotherapy response rate of noninflammatory tumors (Jiang et al., 2020). Ferroptosis agonists increase but ferroptosis antagonists limit radiotherapy efficacy in tumors (Lang et al., 2019). The ferroptosis inducer erastin increases the sensitivity of acute myeloid leukemia cells to chemotherapeutic agents in a RAS-independent manner (Yu et al., 2015). Therefore, biomarkers related to ferroptosis may have the potential to predict the prognosis and therapeutic response.

Long non-coding RNAs (lncRNAs) are a type of nonprotein-coding RNA with a length of >200 nucleotides (Alexander et al., 2010). A large number of lncRNAs are associated with various cancers, exhibiting antitumor or oncogenic functions. Alterations in lncRNA expression and their mutations are involved in tumorigenesis and metastasis (Bhan et al., 2017). Dysregulation of specific lncRNAs is closely related to the ferroptosis process in various malignant tumors (Wu Y et al., 2020). LINC00336 binds the RNA-binding protein ELAVL1, inhibiting ferroptosis in lung cancer (Wang M. et al., 2019). The lncRNA P53RRA promotes ferroptosis and apoptosis by affecting the transcription of several metabolic genes (Mao et al., 2018). However, relatively few studies have assessed the roles of lncRNAs in the ferroptosis process in stomach cancer.

In this study, the potential associations of ferroptosis-related lncRNAs with prognosis and treatment efficacy in patients with stomach cancer were explored. By integrating The Cancer Genome Atlas (TCGA) and the FerrDb databases, a prognostic model based on 10 prognostic ferroptosis-related lncRNAs (*AC009299.2*, *AC012020.1*, *AC092723.2*, *AC093642.1*, *AC243829.4*, *AL121748.1*, *FLNB-AS1*, *LINC01614*, *LINC02485*, *LINC02728*) was constructed, and patients were divided into different risk subgroups based on risk scores. The biological mechanism underlying different prognoses and the functions of these 10 lncRNAs were revealed. Then, the association between these lncRNAs and clinicopathological features was analyzed and several clinical factors related to prognosis were used to construct a nomogram that predicted the overall survival (OS) probability. Next, the association between this model and treatment efficacy was explored. Immune infiltration, immune checkpoint inhibition therapy, and sensitivity to chemotherapeutic drugs were analyzed. We aimed to provide effective biomarkers for predicting the prognosis and therapeutic response of patients with stomach cancer.

## Materials and Methods

### Data Mining and Processing

The transcriptional expression and corresponding clinical data of stomach cancer samples were downloaded from UCSC Xena^1^ (Goldman et al., 2020). The miRNA expression was obtained from TCGA^2^ (Hutter and Zenklusen, 2018). The known 259 ferroptosis-related genes were downloaded from the FerrDb database^3^ (Zhou and Bao, 2020). Samples without clinical information and OS times less than 30 days were deserted. LncRNAs and mRNAs were annotated according to annotated files downloaded from the GENCODE project^4^ (Frankish et al., 2019). Gene expression was transformed into Transcripts Per Kilobase of exon model per Million mapped reads (TPM) format to eliminate the effects of sequencing depth and gene length. Finally, 337 STAD samples were divided into a training cohort and a test cohort by 2:1 in the “caret” R package.

### Construction of a Prognostic Model

Spearman correlation analysis was conducted to screen lncRNAs related to ferroptosis with the condition of “*p*-value <0.01 and absolute values of correlation coefficients ≥0.5”. Firstly, univariate Cox proportional hazards regression (UniCox) analysis was used to screen lncRNAs associated with overall survival. All these lncRNAs were further enrolled into Least absolute shrinkage and selection operator (Lasso) analysis for dimension reduction in the “glmnet” R package. Then, prognostic lncRNAs, as risk signatures, were identified and used to construct a prognostic model in Multivariate Cox proportional hazards regression (MultiCox) analysis. Risk scores were calculated in a linear combination of expression values and regression coefficients of risk signatures. Based on the median value of risk scores in the training cohort, patients could be divided into the high- and low-risk groups. A Sankey plot was plotted to depict the relationship of ferroptosis-related genes, screened lncRNAs associated with prognosis and ferroptosis, and protect or risk roles to OS in the “ggalluvial” R package.

### Assessment and Validation in a Prognostic Model

Considering the specialty of survival status, time-independent ROC curves, along with Area under these ROCs, were done to assess the sensitivity, and specificity of the prognostic model in the “timeROC” R package. Survival probability was compared between the high- and low-risk groups by Kaplan–Meier (KM) survival analysis. The risk score curve, the survival status distribution curve, and the expression heatmap were plotted. In addition, the expression of lncRNA signatures between the high- and low-risk groups was compared with the Mann-Whitney U test. Significance levels were annotated on the right side. All these analyses were conducted in two test cohorts to validate the results. In addition, survival rates of signatures with different expression levels were analyzed in the KM curves, which again indicated their roles to OS.

### Risk Signatures and Clinical Factors

Overall risk scores and expression of these lncRNAs in different clinical subgroups were compared using the Mann-Whitney U test in the test2 cohort. Clinical factors included age, gender, neoplasm histological grade, pathological T, pathological M, pathological N, and tumor stage. *p* < 0.05 was thought to have a significant difference.

### Construction and Assessment of a Nomogram

UniCox analysis was used to identify clinicopathological factors related to prognosis in the test2 cohort, with the screening condition of *p* < 0.05. A nomogram was constructed to predict OS probabilities at 1-, 3-, and 5-years by integrating these prognostic features. The concordance index (C-index) was calculated to assess the performance of this nomogram. And calibration curves at 1-, 3-, 5-years were plotted to assess agreement between the predicted and actual OS rates in the “rms” R package.

### Function Analysis

The Kyoto Encyclopedia of Genes and Genomes (KEGG) pathways analysis was used to elucidate underlying biological mechanisms resulting in differences in the prognoses of two risk subgroups, which was performed in GSEA V4.1.0 software. NOM *p*-value < 0.05, FDR q-value < 0.25, and |NES| > 1 were the cutoff criterion. A ceRNA network was constructed to reveal the functions of lncRNA signatures, which was displayed by Cytoscape V3.7.1 software. Firstly, 171 DEmiRNAs and 4,288 DEmRNAs between the high- and low-risk group in the test2 cohort were identified in the “edger” R package. Next, lncRNA signatures were enrolled into the miRCode database^5^ (Jeggari et al., 2012) to predict corresponding miRNAs, and then the lncRNA-miRNA axes were built by taking intersection with DEmiRNAs. Subsequently, target mRNAs were obtained by integrating DEmRNAs, and the predicted results from TargetScan^6^ (Agarwal et al., 2015), miRDB^7^ (Chen and Wang, 2020), and miRTarBase^8^ (Huang et al., 2020) database. Predicted mRNAs, found in at least 2 databases, were selected. Finally, the GO terms in biological process (BP) and the KEGG pathways were revealed via the “clusterProfiler” R package. *p*-value and q-value were both 0.05 as cutoff values.

### Evaluation of Immune Infiltration

Single-sample Gene Set Enrichment Analysis (ssGSEA) was performed to quantify immune infiltration between two risk groups in the “gsva” R package. The immune activity of 16 immune cells and 13 immune functions was quantified in the “gsva” R package. The higher the ssGSEA score, the stronger the immune activity. The immune difference between the two risk groups was depicted in the heatmap and violin plots. In addition, stromal scores and immune scores for patients with STAD were calculated in the“estimate” R package by applying the ESTIMATE algorithm (Yoshihara et al., 2013). The stromal and immune scores in risk subgroups were compared in violin plots.

### Immune Checkpoint Therapy

Tumor mutation burden (TMB), defined as the number of somatic mutations per 1000,000 bases, was a biomarker that predicted the efficacy of immunotherapy. Tumor mutation data were downloaded from the TCGA database^9^ (Hutter and Zenklusen, 2018), and the TMB between the two risk groups was calculated and compared. Besides, gene mutations of patients with STAD were presented via the “maftools” R package. The top 20 genes with the most mutations were selected. Tumor Immune Dysfunction and Exclusion (TIDE) scores for patients, which also did help to predict immune checkpoint inhibitor (ICI) therapy response, were calculated in the TIDE database^10^ (Fu et al., 2020). The TIDE scores in risk subgroups were compared in violin plots. Finally, the expression of known immune checkpoints between the high- and low-risk groups was compared.

### Sensitivity to Chemotherapy Drugs

The half-maximal inhibitory concentration (IC50) values of chemotherapeutic drugs were predicted in the “pRRophetic” R package. By building statistical models from gene expression and drug sensitivity data in a very large panel of cancer cell lines, this package can predict the chemotherapeutic response from Tumor Gene Expression Levels (Geeleher et al., 2014). We explored 5 common chemotherapeutic drugs for stomach cancer, including mitomycin. C, cisplatin, docetaxel, etoposide, and paclitaxel.

### Statistical Analysis

All data were processed in the R 4.1.0 software. The difference in two distinct subgroups was compared with the Mann-Whitney U test. *p*-value <0.05 was a criterion of statistically significant differences.

## Results

### Data and Corresponding Clinicopathological Features

**FIGURE 1:**
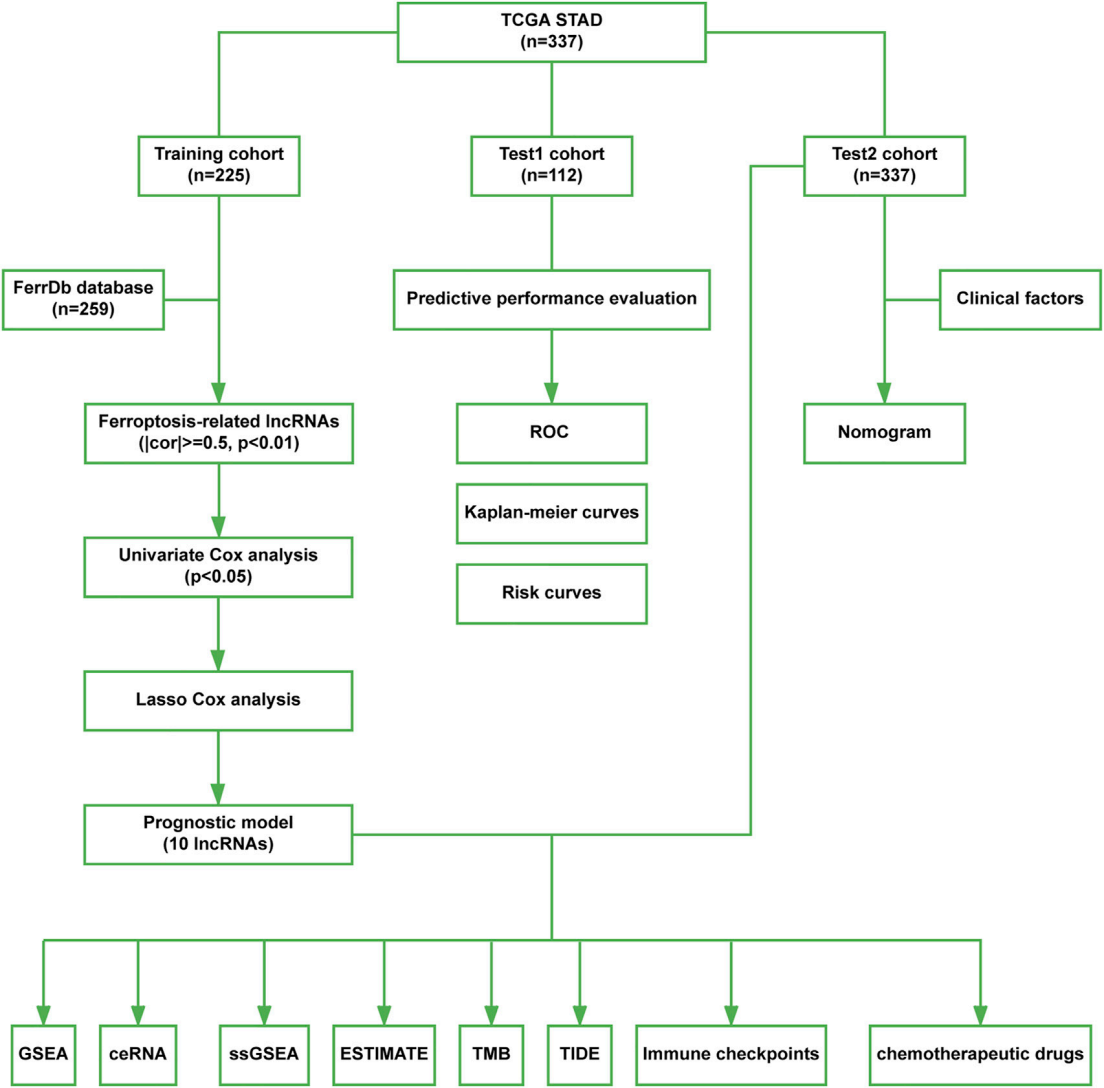
The flow chart in this study.

A flow chart of data process and analysis in this study was depicted (Figure 1). After data preprocessing and partitioning, there were 225 STAD samples in the training cohort, 112 STAD samples in the test1 cohort, and 337 STAD samples in the test2 cohort. Their clinicopathological features were summarized (Table 1).

**TABLE 1:** Clinicopathologic characteristics of patients with STAD in the training cohort and two test cohorts.

	Training cohort	Test cohort	
	TCGA (*n* = 225)	TCGA (*n* = 112)	TCGA (*n* = 337)
Age			
≥65	133	56	189
<65	90	55	145
Gender			
Male	145	73	218
Female	80	39	119
Grade			
G1	6	3	9
G2	82	38	120
G3	131	68	199
GX	6	3	9
T			
T1	10	5	15
T2	47	27	74
T3	109	47	156
T4	56	32	88
TX	3	1	4
M			
M0	202	101	303
M1	14	8	22
MX	9	3	12
N			
N0	64	35	99
N1	63	28	91
N2	40	28	68
N3	50	18	68
NX	7	2	9
Stage			
Stage i	31	14	45
Stage ii	69	38	107
Stage iii	97	40	137
Stage iv	18	16	34
Survival status			
Alive	130	65	195
Dead	95	47	142

### Prognostic Model Construction

**FIGURE 2:**
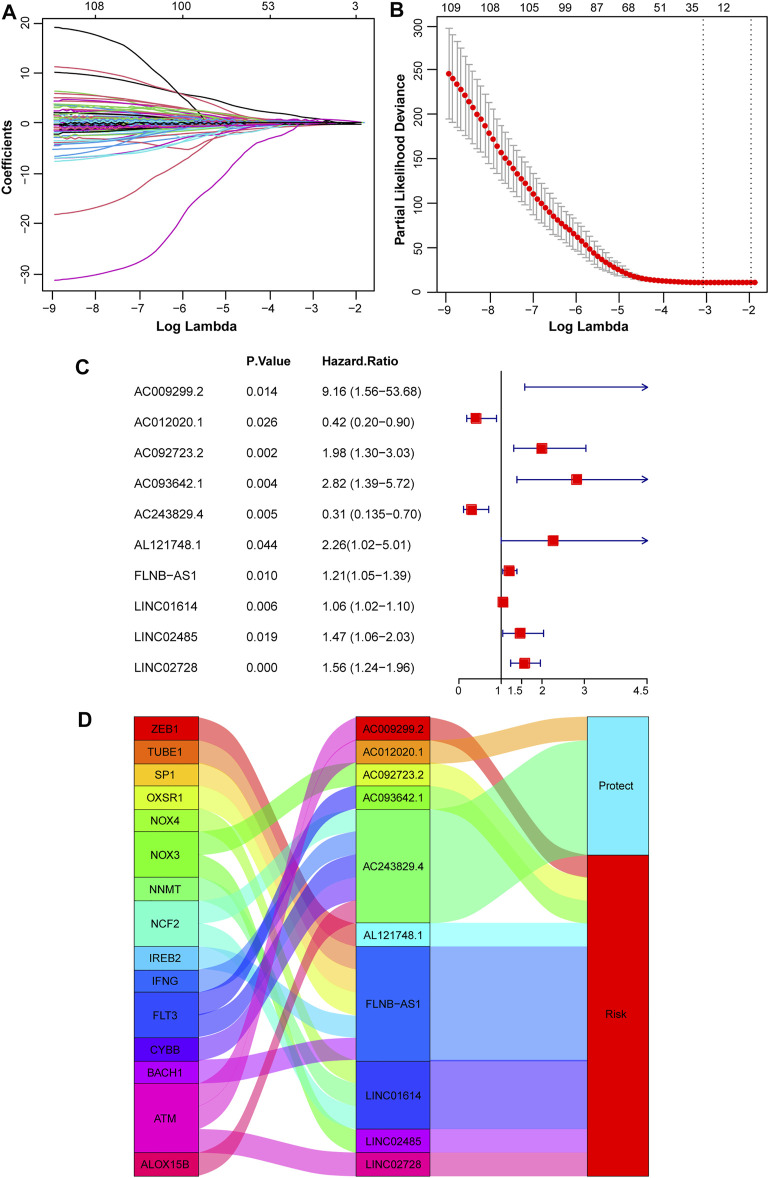
Construction of a prognostic model based on ferroptosis-related lncRNAs. **(A, B)** Variable coefficients and the partial likelihood deviance under different lambda in the Lasso analysis. **(C)** 10 independent prognostic lncRNAs were found *via* the MultiCox regression analysis. **(D)** A Sankey diagram showed the relationship of ferroptosis-related genes, screened 10 lncRNAs, and protect or risk roles in OS.

To identify lncRNAs associated with prognosis and ferroptosis, spearman coefficient, and UniCox analysis were conducted and 109 lncRNAs were identified. Then Lasso analysis was applied to reduce redundancy and simplify models. When partial likelihood deviance was minimal, 32 candidate lncRNAs were obtained as candidates (Figures 2A,B). After MultiCox analysis, 10 ferroptosis-related lncRNAs were identified and used to construct a prognostic model (Figure 2C; Supplementary Table S2). A Sankey diagram was applied to display the relationship of ferroptosis-related genes, lncRNAs in the model, and their roles in OS. *AC012020.1* and *AC243829.4* were protective factors and the others were risk factors for OS (Figure 2D). The formula was shown below:

Risk Score = 2.214344584 * Exp_*AC009299.2*_ − 0.859698908 * Exp_*AC012020.1*_ + 0.68393393 * Exp_*AC092723.2*_ + 1.037748564 * Exp_*AC093642.1*_ − 1.181411845 * Exp_*AC243829.4*_ + 0.815962314 * Exp_*AL121748.1*_ + 0.188412024 * Exp_*FLNB-AS1*_ + 0.058077818 * Exp_*LINC01614*_ + 0.384845713 * Exp_*LINC02485*_ + 0.44150546 * Exp_*LINC02728*_.

### Assessment and Validation

**FIGURE 3:**
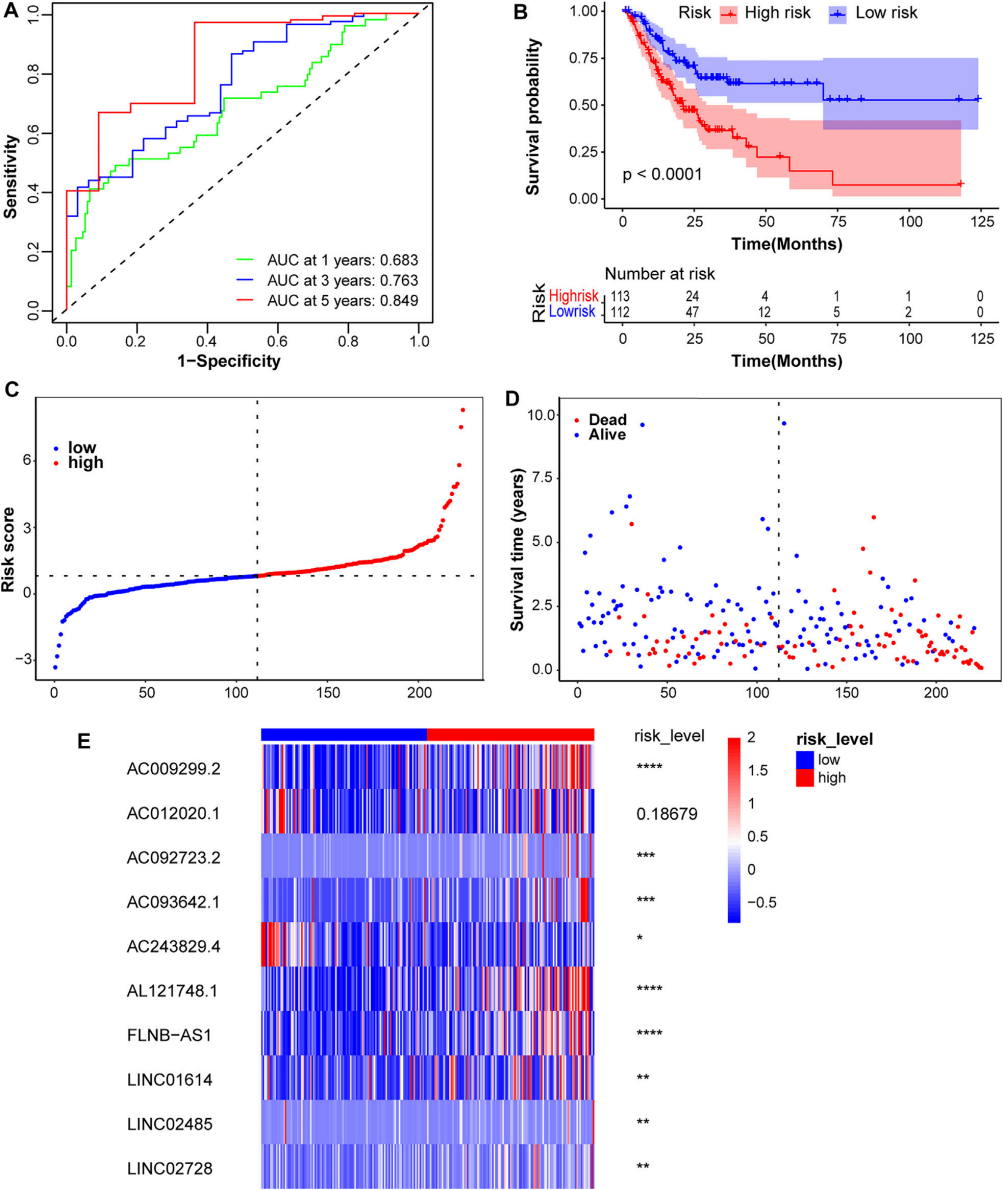
Assessment of the prognostic model in the training cohort. **(A)** Time-independent ROC curves along with their AUC were conducted to assess the sensitivity and specificity. **(B)** The KM survival curves showed a higher survival rate in the low-risk group. **(C)** Risk scores for patients with STAD were presented in ascending order. **(D)** The survival status distribution curve showed a higher proportion of dead patients in the high-risk group. **(E)** The expression heatmap of these 10 lncRNAs was plotted and significance levels were annotated on the right side. ****p < 0.0001, ***p < 0.001, **p < 0.01, *p < 0.05.

Risk scores for all patients with STAD were calculated, and patients were divided into high- and low-risk groups, based on the median score of 0.815527251 in the training cohort. The predictive sensitivity and specificity of this prognostic model were assessed by constructing time-independent ROC curves. The AUCs of ROC curves at 1-, 3-, and 5-years were 0.683, 0.763, and 0.849, respectively, which indicated good performance (Figure 3A). The KM curve showed that the high-risk subgroup had a shorter overall survival time (Figure 3B). Risk scores for patients with STAD were presented in ascending order (Figure 3C). The survival distribution curve showed a higher proportion of dead patients in the high-risk group and a lower proportion of dead patients in the low-risk group (Figure 3D). Compared with the low-risk group, the expression levels of eight lncRNA signatures were significantly upregulated in the high-risk group, while *AC243829.4* expression was reversed and *AC012020.1* tended to be downregulated (Figure 3E). The same analyses were conducted to validate in the test1 cohort containing new external samples and the test2 cohort containing all STAD samples. Similar results were obtained, except that the differences in the expression of several lncRNAs were not significant in the test1 cohort, potentially due to its small sample size (Supplementary Figures S1, S2). The KM survival analysis of 10 lncRNAs showed that higher expression of *AL121748.1*, *LINC01614*, and *AC009299.2* was associated with a shorter OS probability, which again indicated their risk roles (Supplementary Figure S3).

### Signatures and Clinical Factors

Risk scores for different clinical subgroups were compared in the test2 cohort to elucidate the association between the prognostic model and clinical factors. Patients with distant metastases had higher risk scores, and patients with pathological T3 or T4 tended to higher risk scores. However, no significant differences were observed in other clinical subgroups (Supplementary Figures S4, S5A). KM survival analyses of pathological M subgroups were conducted to determine whether clinical factors affected the prognostic performance of this model. Patients with higher risk scores in the pathological M0 subgroup still had shorter overall survival time (Supplementary Figure S5B). A statistically significant difference was not observed, but a trend toward a difference was identified in the pathological M1 group, potentially because of the limited number of 22 samples (Supplementary Figure S5C). These analyses indicated that risk scores predict OS independently. Gene expression levels in different clinical subgroups were analyzed to further explore the potential link between the signatures and clinicopathological features. The expression of four lncRNAs was associated with age (Supplementary Figure S6A), but not with gender (Supplementary Figure S6B). *AC093642.1*, *AC243829.4*, *AL121748.1*, *LINC01614*, and *LINC02728* were expressed at higher levels in the higher neoplasm histological grade group (Supplementary Figure S6C). Only the expression of *AC009299.2* and *FLNB-AS1* in the tumor stage subgroups was significantly different (Supplementary Figure S6D). The expression of *AC009299.2* and *AL121748.1* in the pathological T subgroups was significantly different (Supplementary Figure S6E), but no differences in the pathological M subgroups (Supplementary Figure S6F). Compared with the pathological N0 or N1 group, the expression of *AC009299.2*, *AC093642.1*, *AL121748.1*, and *FLNB-AS1* was significantly different in the pathological N2 or N3 group (Supplementary Figure S6G). The results from analyses between 10 signatures and clinical features again indicated roles for some lncRNAs as risk factors, especially *AC009299.2*.

### Nomogram Construction and Assessment

**FIGURE 4:**
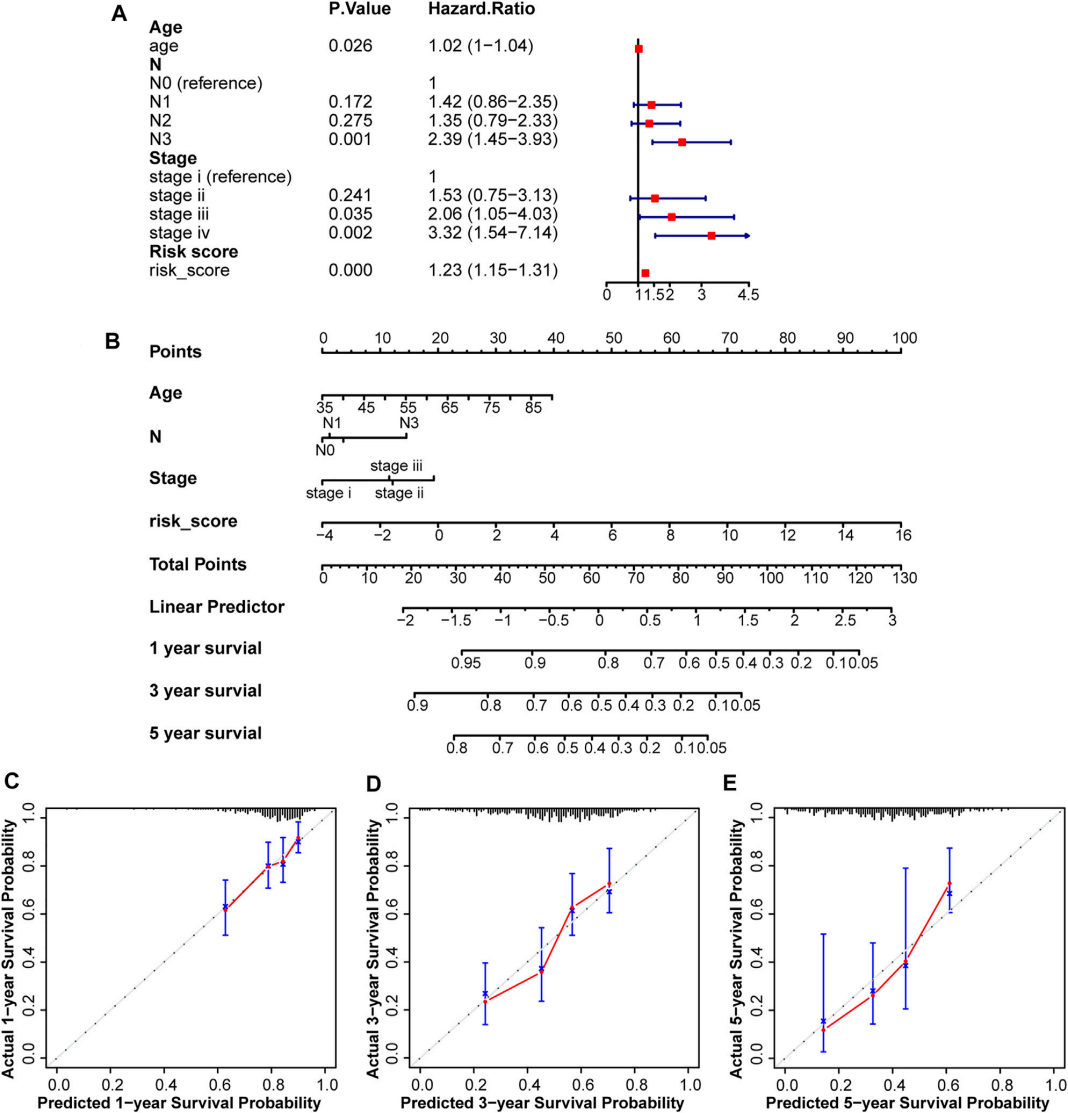
Construction and assessment of a nomogram in the test2 cohort. **(A)** Four factors including age, pathological N, tumor Stage, and risk score, were found to be related to prognosis. **(B)** A nomogram was constructed to predict overall survival rates at 1-, 3-, and 5-years. **(C–E)** Calibration curves were plotted to assess agreement between the predicted and actual OS rates.

Using the prognostic factors, a nomogram was built to predict the survival rates of patients with STAD. The UniCox analysis identified several factors affecting prognosis, including age, pathological N, tumor stage, and risk score (Figure 4A). This nomogram was used to predict the 1-, 3-, and 5-years overall survival rates of patients with STAD (Figure 4B). The C-index was 0.67118161, and the calibration curves at 1-, 3-, and 5-years showed a good match between the predicted and actual OS rates (Figures 4C–E).

### Functional Analysis

**FIGURE 5:**
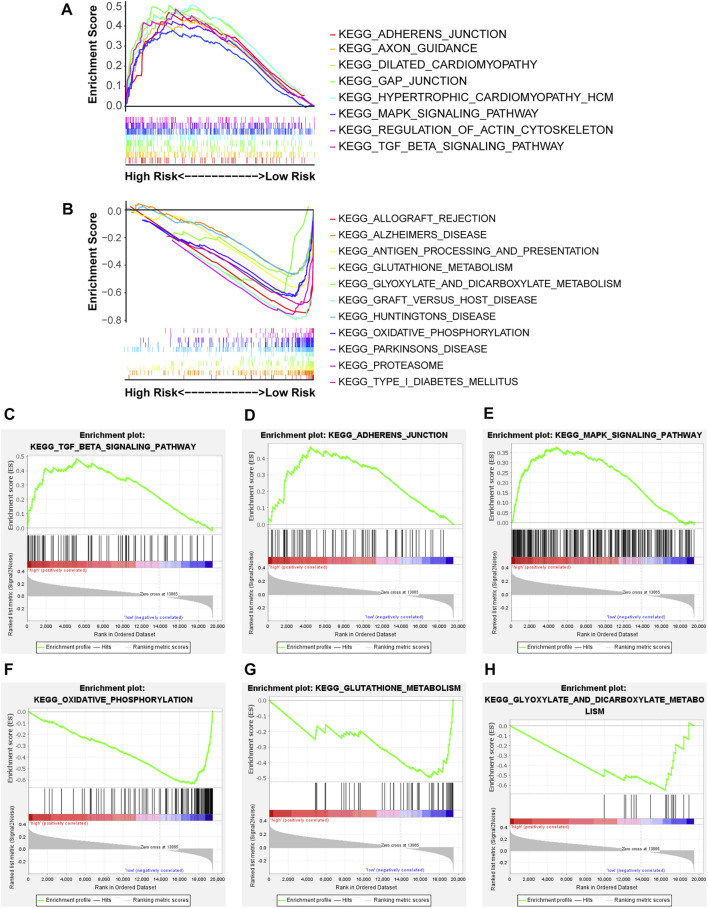
The enriched KEGG pathways were revealed in the two risk groups *via* GSEA software. NOM *p*-value < 0.05, FDR q-value < 0.25, and |NES| > 1 were the cutoff criterion. **(A,B)** Distinct pathways enriched in the high- and low-risk groups, **(C)** Enrichment plot: KEGG_TGF_BETA_SIGNALING_PATHWAY, **(D)** Enrichment plot: KEGG_ADHERENS_JUNCTION, **(E)** Enrichment plot: KEGG_MAPK_SIGNALING_PATHWAY, **(F)** Enrichment plot: KEGG_OXIDATIVE_PHOSPHORYLATION, **(G)** Enrichment plot: KEGG_GLUTATHIONE_METABOLISM, and **(H)** Enrichment plot: KEGG_GLYOXYLATE_AND_DICARBOXYLATE_METABOLISM.

**FIGURE 6:**
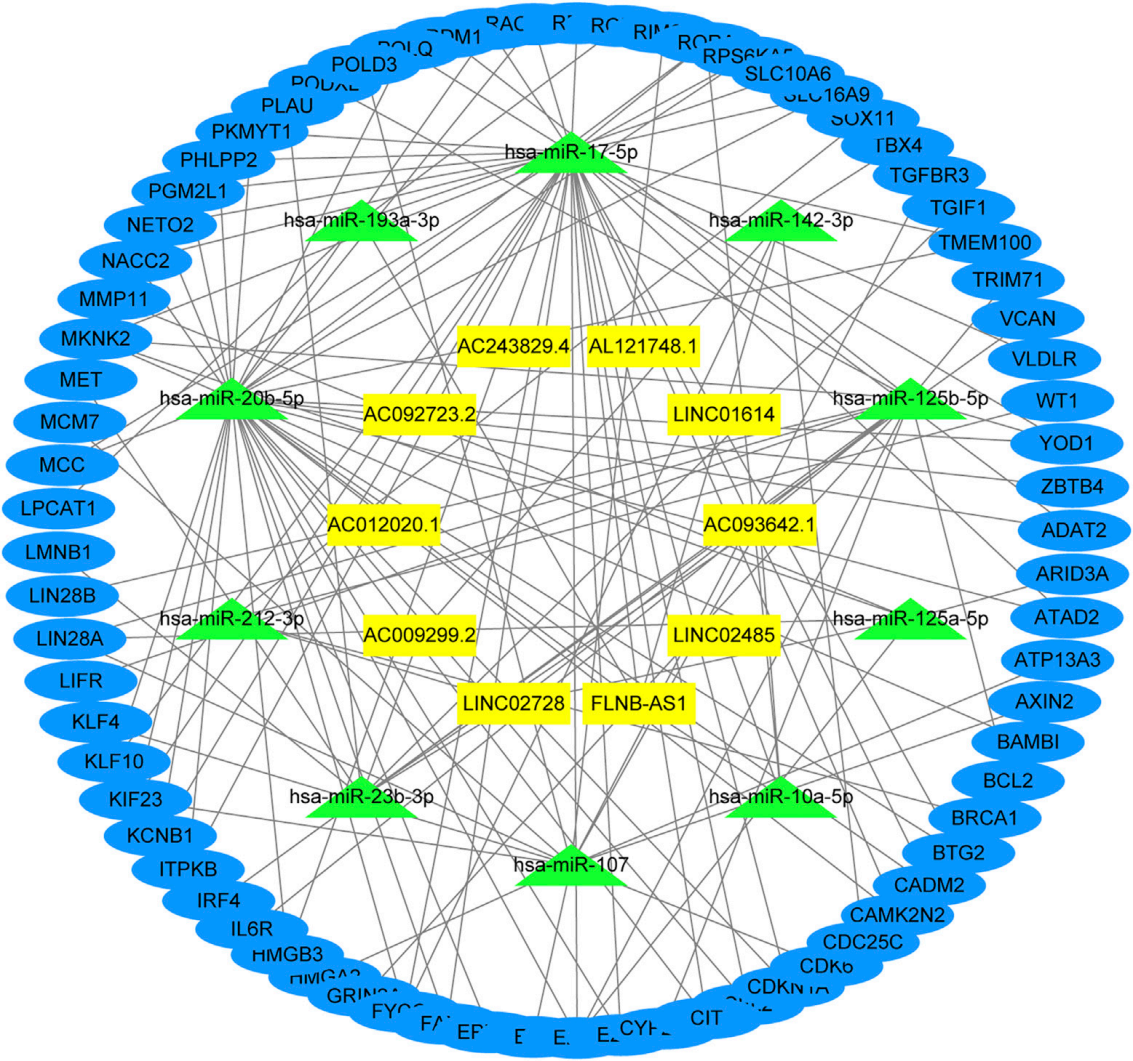
Function analysis of these 10 lncRNAs. The lncRNA-miRNA-mRNA network was constructed by integrating differential genes and predicted results from miRCode, TargetScan, miRDB, and miRTarBase database.

The underlying biological mechanisms resulting in differences in the prognoses of the two risk subgroups were elucidated by conducting KEGG pathway analysis with GSEA software in the test2 cohort. Genes in the low-risk group were enriched in multiple pathways, including oxidative phosphorylation, glutathione metabolism, and glyoxylate and dicarboxylate metabolism. Genes in the high-risk group were significantly enriched in the TGF-beta signaling pathway, adherens junction, and MAPK signaling pathway, among others (Figure 5). A ceRNA network was constructed, and functional enrichment analysis based on target mRNAs was conducted to reveal the functions of these 10 lncRNAs. LncRNA-miRNA-mRNA axes were displayed (Figure 6; Supplementary Table S3). Five lncRNAs (*AC093642.1*, *LINC02485*, *FLNB-AS1*, *LINC02728,* and *AC009299.2*) regulated the expression of 73 mRNAs through the corresponding 10 miRNAs, thus participating in a variety of biological processes. Functional enrichment analysis identified GO terms in BP and KEGG pathways (Supplementary Table S4). The top 20 results were shown (Supplementary Figure S7).

### Evaluation of Immune Infiltration

**FIGURE 7:**
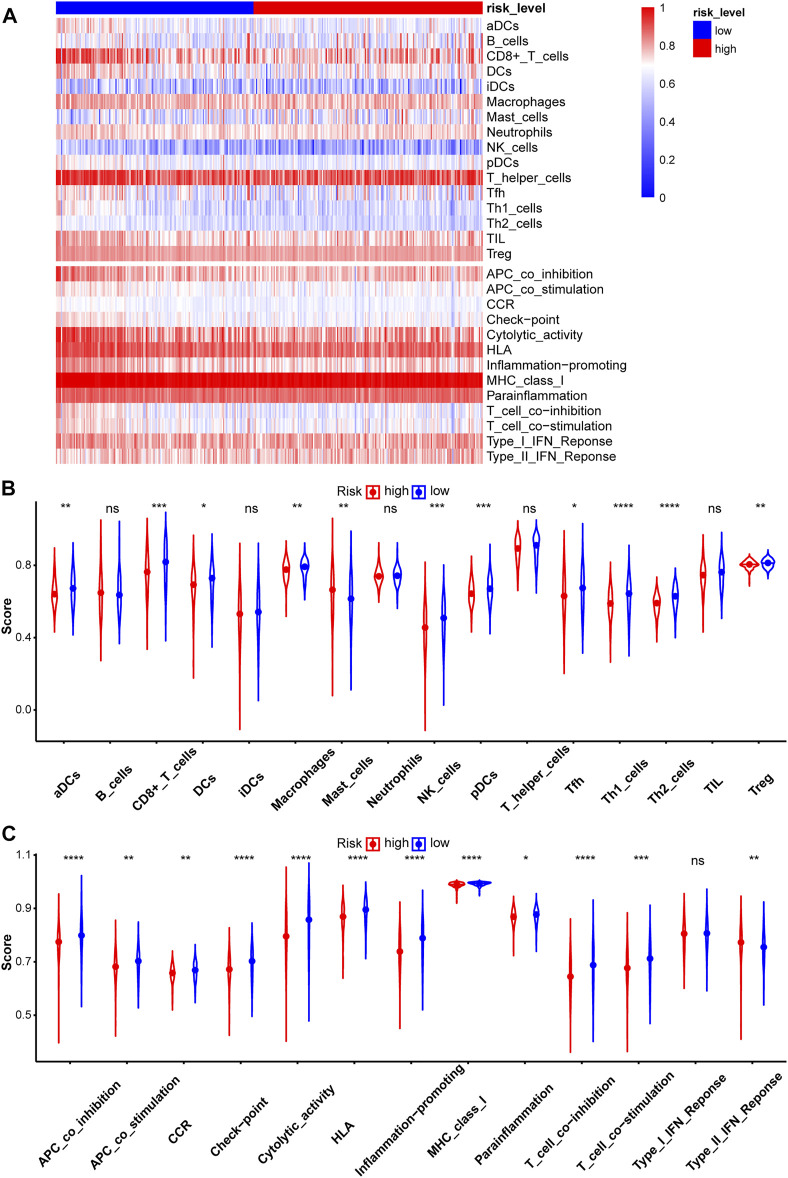
Evaluation of Immune infiltration between two risk groups in the test2 cohort. **(A)** The heatmap of ssGSEA scores in patients with STAD was plotted to quantify the immune activity of immune cells and immune functions. The ssGSEA scores of 16 immune cells **(B)** and 13 immune functions **(C)**.

**FIGURE 8:**
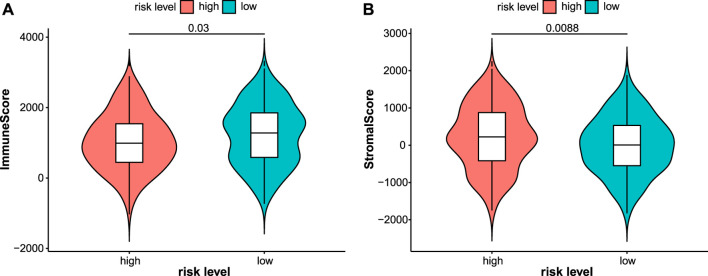
Immune and stromal scores were calculated by the ESTIMATE algorithm in the test2 cohort. Patients in the low-risk group had higher immune scores **(A)** while lower stromal scores **(B)**.

Immunotherapy is a new treatment for stomach cancer that may improve the antitumor ability in patients by activating their immune systems. However, not all patients with gastric cancer are suitable for immunotherapy, suggesting that identifying these patients is vital. Here, the association between the immune landscape and the prognostic model was explored to determine whether the risk score helps to identify patients who would possibly benefit from immunotherapy. Immune infiltration was compared between the two risk groups using ssGSEA and the ESTIMATE algorithm. A heatmap of ssGSEA scores for 16 immune cells and 13 immune functions was plotted (Figure 7A). The higher the ssGSEA score, the stronger the immune activity. Compared with the high-risk group, the immune activities of most innate immune cells (aDCs, DCs, macrophages, NK cells, and pDCs) and adaptive immune cells (CD8^+^ T, Thf, Th1, Th2, and Treg cells) were higher in the low-risk group (Figure 7B). Similar results were obtained for immune functions, such as checkpoint, cytolytic activity, and type II IFN response (Figure 7C). Immune scores and stromal scores of STAD samples were calculated using the ESTIMATE algorithm. Higher immune scores and lower stromal scores were observed in the low-risk group (Figure 8). Considering the immune-activating environment, particularly the higher immune activity of the “checkpoint”, and higher immune scores of the low-risk group, the response to immunotherapy would likely differ between the two risk groups. Hence, immune checkpoint inhibitor therapy was analyzed next.

### Immune Checkpoint Inhibitor Therapy

**FIGURE 9:**
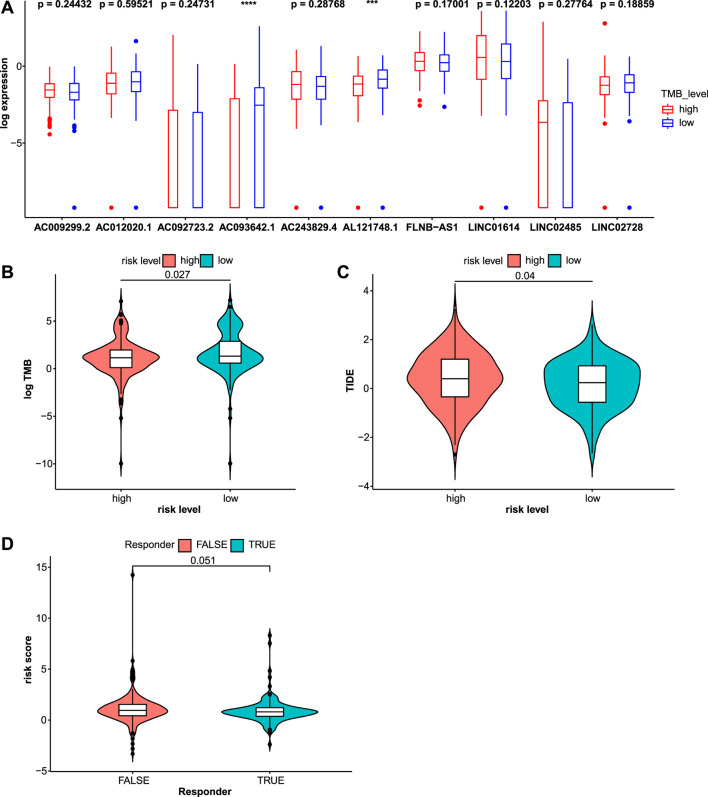
Immune checkpoint inhibitor (ICI) therapy response was assessed through the TMB and TIDE scores in the test2 cohort. **(A)** The expression values of these 10 lncRNAs were compared in different TMB levels. **(B)** The TMB was significantly distinct in the high- and low-risk groups. **(C)** Patients in the high-risk group had higher TIDE scores indicating a higher chance of immune evasion. **(D)** Risk scores for patients who responded to ICI therapy and those who did not were compared.

**FIGURE 10:**
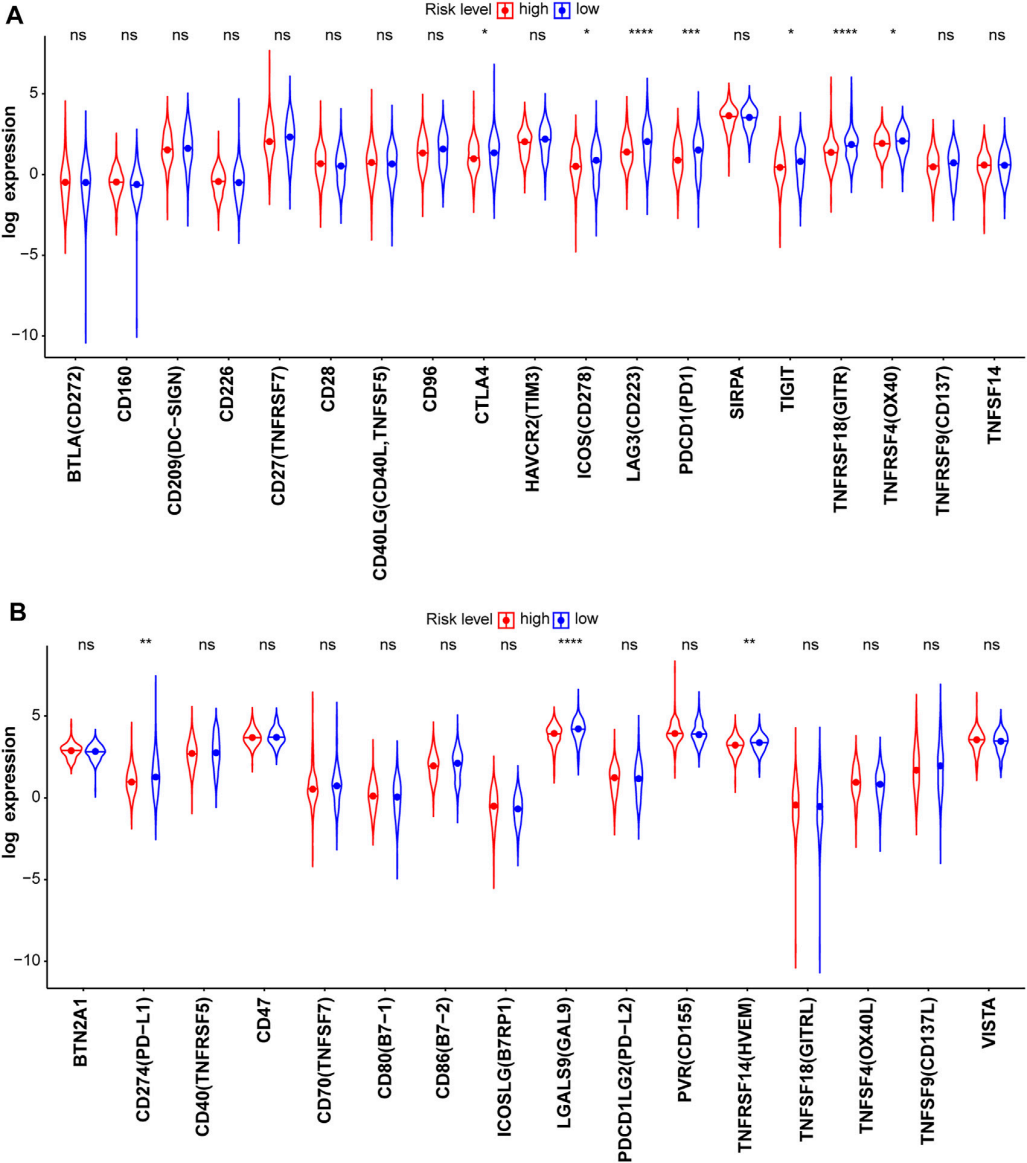
Known immune checkpoints **(A)** and their ligands **(B)** in the test2 cohort.

The TMB is a potential biomarker for selecting patients who may respond to immune checkpoint inhibitor therapy (Chan et al., 2019). A greater benefit of anti-PD-L1 monoclonal antibody was observed for patients with a high TMB (Peters et al., 2017). The TMB was calculated and compared between the two risk groups (Figure 9B). Patients in the low-risk group had a higher TMB, indicating that they may benefit from ICI therapy. The association between the expression of 10 lncRNAs and the TMB was analyzed. Higher TMBs were observed in patients with lower expression of *AC093642.1* and *AL121748.1* (Figure 9A). In addition, gene mutations in STAD samples based on risk levels were presented in detail (Supplementary Figure S8). The top three genes were *TTN*, *TP53*, and *MUC16*. Genes exhibited different mutation ratios including *KMT2D* [16 of 176 (9.1%) *vs.* 34 of 153 (22.2%)], *PIK3CA* [15 of 176 (8.5%) *vs.* 32 of 153 (20.9%)], *ARID1A* [35 of 176 (19.9%) *vs.* 47 of 153 (30.7%)], *HMCN1* [19 of 176 (10.8%) *vs.* 32 of 153 (20.9%)], and *TTN* [75 of 176 (42.6%) *vs.* 79 of 153 (51.6%)]. TIDE scores were calculated to predict the ICI response, which were used to simulate two mechanisms of tumor immune evasion: inducing T cell dysfunction and preventing T cell infiltration (Jiang et al., 2018; Fu et al., 2020). Patients with lower TIDE scores had a lower chance of immune evasion and received more benefits from ICI therapy. The violin plot showed that patients in the low-risk group had lower TIDE scores (Figure 9C). Patients with STAD who responded to immune checkpoint inhibitors had lower risk scores (Figure 9D). Next, we explored the relationship between risk scores and common immune checkpoints. Compared with the high-risk group, the expression of some immune checkpoints was significantly higher in the low-risk group (Figure 10).

### Chemotherapy Efficacy Related to the Risk Score

**FIGURE 11:**
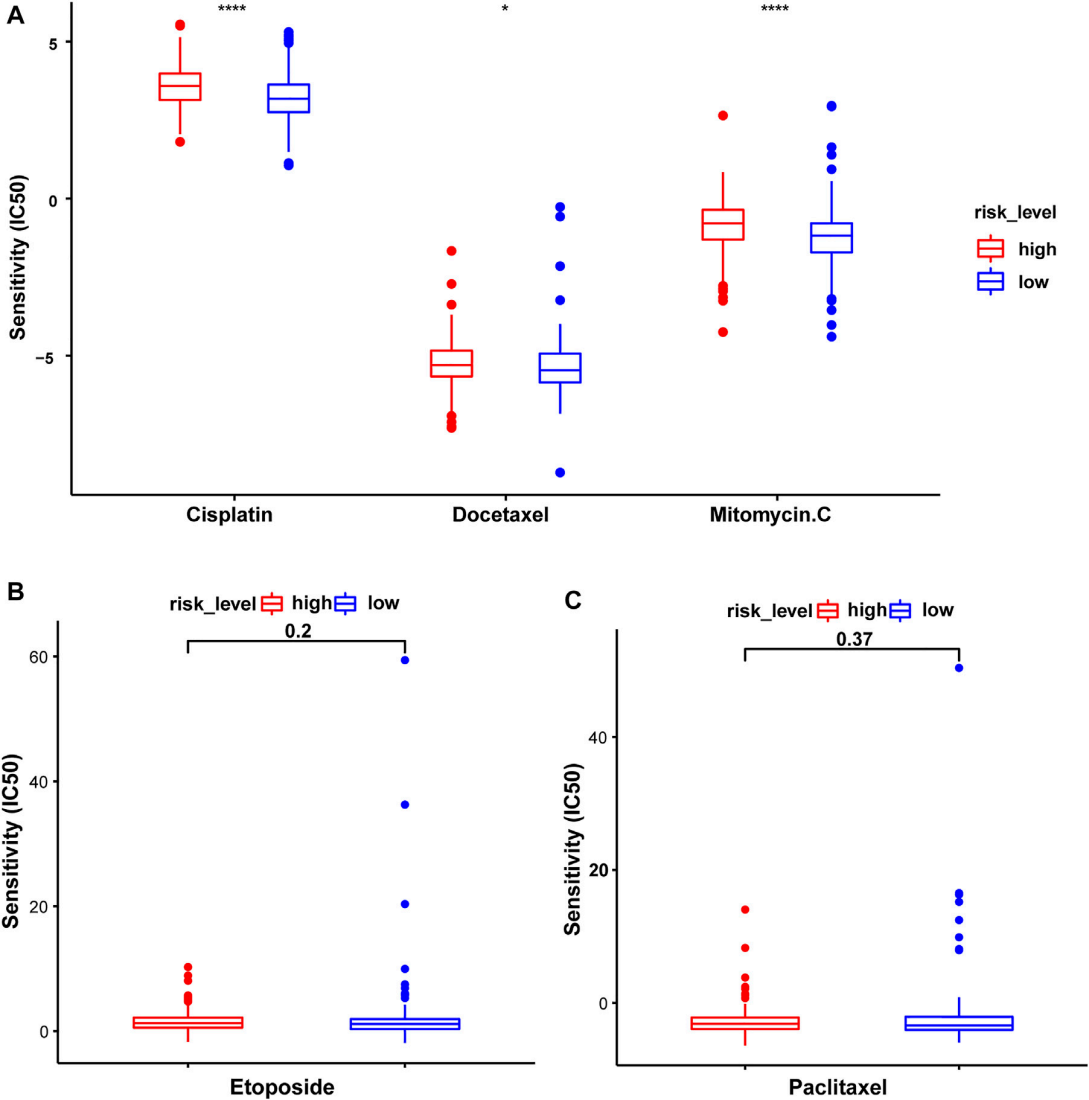
The sensitivity to common chemotherapeutic drugs of patients with STAD in the test2 cohort. **(A)** Cisplatin, Docetaxel, and Mitomycin.C. **(B)** Etoposide. **(C)** Paclitaxel.

The association between this prognostic model and the efficacy of chemotherapeutic drugs was analyzed. The IC50 values of common chemotherapeutic drugs were predicted and compared between the high- and low-risk groups. Patients in the low-risk group had significantly lower IC50 values and were more sensitive to cisplatin, docetaxel, and mitomycin.C. However, no differences in sensitivity to etoposide and paclitaxel were observed (Figure 11).

## Discussion

Ferroptosis is involved in physical conditions or various diseases including cancers (Mou et al., 2019). In recent years, the use of ferroptosis in cancer treatment has attracted great attention. Ultrasmall silica nanoparticles induce ferroptosis and suppress tumor growth, suggesting their therapeutic potential (Kim et al., 2016). The imbalance between the transcription factors HIC1 and HNF4A, regulating ferroptosis up-regulated factors (FUF), and ferroptosis down-regulated factors (FDF), respectively, may help treat liver cancer (Zhang et al., 2019). In lung adenocarcinoma, STK11/KEAP1 commutation leads to resistance to pharmacologically induced ferroptosis and high expression of ferroptosis-protective genes, which is associated with early death and aggressive tumor development (Wohlhieter et al., 2020). These results indicate that the induction of ferroptosis in cancer cells can effectively improve the prognosis and enhance treatment efficacy. Hence, ferroptosis-related biomarkers, which are reliable predictors of the prognosis and treatment response, have been developed for a wide range of malignant tumors (Liu Y. et al., 2020; Wu G. et al., 2020; Zhuo et al., 2020; Jiang et al., 2021). A ferroptosis-related 15-gene signature of LUAD is built and can accurately predict the prognosis (Zhang et al., 2021). A novel ferroptosis-related gene signature for prognostic prediction in patients with glioma is built and the association between these genes and immune checkpoint molecules is revealed (Chen Z. et al., 2021). Considering the important role of lncRNAs in epigenetic regulation, transcriptional regulation, post-transcriptional regulation, and protein-coding gene regulation, we developed a reliable biomarker by integrating ferroptosis and lncRNAs. In our study, a prognostic model with good predictive performance based on 10 ferroptosis-related lncRNAs was constructed and patients with stomach cancer were divided into two risk groups based on risk scores. Patients in the low-risk group had a longer overall survival time than those in the high-risk group.

Underlying biological mechanisms, which resulted in prognostic differences, were revealed by GSEA. Metabolic pathways, including oxidative phosphorylation, glutathione metabolism, and glyoxylate and dicarboxylate metabolism, were enriched in the low-risk group. The Warburg effect shows that the energy needed for cellular processes is primarily generated through mitochondrial oxidative phosphorylation (OXPHOS) and aerobic glycolysis, in normal differentiated cells and most cancer cells, respectively (Vander Heiden et al., 2009). Compared with oxidative phosphorylation, aerobic glycolysis is less efficient at generating adenosine 5′-triphosphate (ATP). Intestinal gastric carcinomas, but not precancerous stages, are often characterized by loss of OXPHOS complex I, acting as tumor suppressors, and this pathological phenomenon occurs independently of *Helicobacter pylori* infection (Feichtinger et al., 2017). Glutathione metabolism and ferroptosis are closely related. The glutathione/GPX4-independent axis suppresses ferroptosis, and GPX4 protects cells from ferroptosis by decreasing phospholipid peroxides via glutathione (Stockwell et al., 2020). In addition, glutathione (GSH) metabolism plays a beneficial or pathogenic role in a variety of malignant tumors and excess GSH promotes tumor progression and metastasis (Bansal and Simon, 2018). Altered glyoxylate and dicarboxylate metabolism are associated with the chromosomal instability status in gastric cancer (Tsai et al., 2018). Genes in the high-risk group were significantly enriched in the TGF-beta signaling pathway, adherens junction, and MAPK signaling pathway, among others. The transforming growth factor (TGF)-β signaling pathway has a dual function and pleiotropic nature. This pathway has tumor suppressor functions in normal and early-stage cancer cells, including cell-cycle arrest and apoptosis, but has cancer-promoting functions in late-stage cancer cells, including metastasis and chemoresistance (Colak and Ten Dijke, 2017; Seoane and Gomis, 2017). Activation of transforming growth factor-beta 1 Signaling in fibroblasts increases the motility and invasiveness of gastric cancer cells (Ishimoto et al., 2017). The TGF-β receptor inhibitor, LY2109761, increases radiosensitivity in GC by regulating the TGF-β/SMAD4 signaling pathway (Yang et al., 2019). Mitogen-activated protein kinases (MAPKs) include three major subfamilies: extracellular-signal-regulated kinases (ERK MAPKs), c-Jun N-terminal kinases, stress-activated protein kinases (JNKs or SAPKs), and MAPK14 (Fang and Richardson, 2005).

Relatively little research has been conducted on these 10 lncRNAs at present. *LINC01614* is identified as an oncogenic lncRNA that promotes proliferation and migration in gastric cancer (Chen Y. et al., 2021). *LINC01614* promotes FOXP1 expression by inhibiting miR-217, which ultimately stimulates the development of LUAD (Liu et al., 2018). Upregulation of *LINC01614*, induced by SP1, promotes malignant glioma progression by modulating the miR-383/ADAM12 axis (Wang et al., 2020). These studies imply that *LINC01614* is a factor contributing to an unfavorable prognosis of various cancers, consistent with our results. The expression levels of *FLNB-AS1* are positively correlated with the survival probability of patients with breast cancer and *FLNB-AS1* may be a potential diagnostic or prognostic marker of tamoxifen resistance (Zhang X. et al., 2020). *AL121748.1* may be involved in multiple metabolic processes, such as amino acid, lipid, and glucose metabolism in cirrhotic HCC (Ma and Deng, 2019). By constructing a ceRNA network, the functions of some lncRNAs were explored. The GO analysis showed that these lncRNAs were involved in cell cycle regulation, especially the G1/S phase transition, which was related to the transition from RNA and ribosome synthesis to the reproduction of genetic material. The KEGG analysis showed that these lncRNAs were involved in several pathways, including microRNAs in cancer, the cell cycle, the PI3K−Akt signaling pathway, and various cancers. However, the functions mentioned above required further biological verification.

Using current standard therapies, the prognosis of patients with advanced-stage gastric cancer remains poor (Zhao et al., 2019). Immunotherapy, an innovative approach, is developed to improve the survival rate of patients with various cancers, such as lung cancer, gastric cancer, and breast cancer. In this study, immune infiltration was assessed using ssGSEA and the ESTIMATE algorithm. The abundance of several innate immune cells and adaptive immune cells, such as CD8^+^ T cells, DCs, pDCs, and Tregs, was lower in the high-risk subgroup. Patients with advanced non-small-cell lung cancer presenting with greater CD8^+^ T cell infiltration exhibit a superior treatment response to pembrolizumab, an anti-PD-1 drug (Garon et al., 2019). Dendritic cells (DCs), which are antigen-presenting cells, often eliminate tumors by stimulating naive T cell differentiation (Fu et al., 2021). HIF-1α inhibits plasmacytoid DC (pDC) differentiation, leading to tumor progression (Labiano et al., 2015). Treg cells are key subsets of effector T cells with strong immunosuppressive effects. The immune activity of most of the immune functions was higher in the low-risk subgroup, such as checkpoint, cytolytic activity, and type II IFN response. The immune cytolytic activity score reflects antitumor immunity and predicts clinical outcomes of patients with GC (Hu et al., 2021). Type II IFN (IFN-γ) is a critical driver of programmed death ligand-1 (PD-L1) expression in cancer and host cells (Ayers et al., 2017). These results showed that patients in the high-risk group were immunosuppressed compared to the other group.

Considering the immune-activating environment and higher immune scores in the low-risk group, we speculated a potentially significant difference in immunotherapy efficacy in the two subgroups. Thus, ICI therapy was studied in detail. The immune checkpoint inhibitor response was assessed by calculating the TMB and TIDE scores. The results showed higher TMB and lower TIDE scores for the low-risk group, indicating that these patients might receive a greater benefit from ICI therapy. Common immune checkpoints, such as PD1, PD-L1, and CTLA4, were expressed at high levels in the low-risk group, which validated this speculation. Pembrolizumab, an anti-PD-1 drug, has shown manageable safety and promising activity in patients with advanced gastric or gastroesophageal junction cancer who have received at least 2 lines of treatment (Fuchs et al., 2018; Smyth et al., 2020). Nivolumab, an antibody inhibitor of programmed death-1 (PD-1), is approved as an option for third- or later-line treatment of advanced gastric/gastroesophageal junction (G/GEJ) cancer (Kang et al., 2017; Janjigian et al., 2018; Boku et al., 2019). In addition, the genetic mutation waterfall diagram showed that *KMT2D*, *PIK3CA*, *ARID1A*, *HMCN1*, and *TTN* mutations were enriched in the low-risk group. *KMT2D* mutant cells show higher protein turnover and IFNγ-stimulated antigen presentation and both mice and human *KMT2D* mutant tumors show increased immune infiltration (Wang G.et al., 2020). The *PIK3CA* mutation may alter the levels of PD-1, PD-L1, and PD-L2 (Cho et al., 2019; Liu J. et al., 2020). The frequency of *TTN* mutations is significantly positively correlated with the objective response rate of patients receiving anti-PD1/PD-L1/CTLA-4 monotherapy (Jia et al., 2019). In summary, risk scores based on 10 ferroptosis-related lncRNAs could help screen patients with stomach cancer who might benefit from immunotherapy.

Accumulating studies have shown that ferroptosis and chemotherapy are inseparable. Erastin, an inducer of ferroptosis, enhances the sensitivity to chemotherapy, and radiotherapy, suggesting a promising future application in cancer therapy (Zhao et al., 2020). Cisplatin and paclitaxel promote miR-522 secretion which decreases the accumulation of lipid-ROS by suppressing ALOX15 expression and ultimately results in chemoresistance in gastric cancer (Zhang H. et al., 2020). In our study, risk stratification based on 10 ferroptosis-related lncRNAs was correlated with the response to several common chemotherapeutic drugs. Patients with stomach cancer in the low-risk group experienced greater survival benefit from chemotherapy with mitomycin. C, cisplatin, and docetaxel. These findings might be helpful to guide the selection of personalized chemotherapy drugs.

Undoubtedly, there were some limitations in this study. Firstly, the sample size was relatively limited. To verify the modeling results, the TCGA cohort was split, which destroyed the model efficiency to a certain extent. Secondly, clinical information was deficient, which resulted in limited analysis related to important clinical factors. Finally, all results were obtained by statistical analysis, biological tests were needed further to verify.

## Data Availability

The original contributions presented in the study are included in the article/Supplementary Material, further inquiries can be directed to the corresponding authors.

## Footnotes

1
http://xena.ucsc.edu/.

2
https://portal.gdc.cancer.gov/.

3
http://www.zhounan.org/ferrdb/.

4
https://www.gencodegenes.org/.

5
http://mircode.org/.

6
http://www.targetscan.org/vert_72/.

7
http://mirdb.org/.

8
https://mirtarbase.cuhk.edu.cn/∼miRTarBase/miRTarBase_2019/php/index.php.

9
https://portal.gdc.cancer.gov/.

10
http://tide.dfci.harvard.edu/.

## Supplementary Material

Supplementary Figure 1 Validation of the prognostic model in the test1 cohort. **(A)** Time-independent ROC curves along with their AUC were conducted to assess the sensitivity and specificity. **(B)** The KM survival curves showed a higher survival rate in the low-risk group. **(C)** Risk scores for stomach patients with STAD were presented in ascending order. **(D)** The survival status distribution curve showed a higher proportion of dead patients in the high-risk group. **(E)** The expression heatmap of these 10 lncRNAs was plotted and significance levels were annotated on the right side. *****p* < 0.0001, ****p* < 0.001, ***p* < 0.01, **p* < 0.05.

Supplementary Figure 2 Validation of the prognostic model in the test2 cohort. **(A)** Time-independent ROC curves along with their AUC were conducted to assess the sensitivity and specificity. **(B)** The KM survival curves showed a higher survival rate in the low-risk group. **(C)** Risk scores for patients with STAD were presented in ascending order. **(D)** The survival status distribution curve showed a higher proportion of dead patients in the high-risk group. **(E)** The expression heatmap of these 10 lncRNAs was plotted and significance levels were annotated on the right side. *****p* < 0.0001, ****p* < 0.001, ***p* < 0.01, **p* < 0.05.

Supplementary Figure 3 The KM survival curves of these 10 lncRNAs with different expression levels were plotted. Based on their median values, lncRNAs were divided into high and low expression groups.

Supplementary Figure 4 The association between risk scores and clinicopathological factors in the test2 cohort.

Supplementary Figure 5
**(A)** Risk scores for patients with or without distant metastases were compared. The KM survival analyses in two pathological M subgroups were conducted, including **(B)** patients without distant metastases (M0) and **(C)** patients with distant metastases (M1).

Supplementary Figure 6 The association between the expression of lncRNAs and clinicopathological factors in the test2 cohort. *****p* < 0.0001, ****p* < 0.001, ***p* < 0.01, **p* < 0.05.

Supplementary Figure 7 Functional analysis of these 10 lncRNAs. The top 20 results of GO terms in BP **(A, B)** and KEGG pathways **(C, D)** were shown. P-value and q-value were both 0.05 as cutoff values.

Supplementary Figure 8 Gene mutations based on risk levels in the test2 cohort. The top 20 genes were presented.
