# Corrigendum: Prognostic ferroptosis-related lncRNA signatures associated with immunotherapy and chemotherapy responses in patients with stomach cancer

**DOI:** 10.3389/fgene.2022.973325

**Published:** 2022-07-22

**Authors:** Donglin Lai, Lin Tan, Xiaojia Zuo, DingSheng Liu, Deyi Jiao, Guoqing Wan, Changlian Lu, Dongjie Shen, Xuefeng Gu

**Affiliations:** ^1^ Shanghai Key Laboratory of Molecular Imaging, Zhoupu Hospital, Shanghai University of Medicine and Health Sciences, Shanghai, China; ^2^ School of Health Science and Engineering, University of Shanghai for Science and Technology, Shanghai, China; ^3^ The Affiliated Zhuzhou Hospital Xiangya Medical College CSU, Zhuzhou, China; ^4^ School of Pharmacy, Shanghai University of Medicine and Health Sciences, Shanghai, China; ^5^ Department of General Surgery, Ruijin Hospital Luwan Branch, Shanghai Jiaotong University School of Medicine, Shanghai, China

**Keywords:** stomach cancer, ferroptosis, prognostic, lncRNA, immunotherapy, chemotherapy

In the published article, an author name was incorrectly written as Donlin Lai. The correct spelling is Donglin Lai.

The authors apologize for this error and state that this does not change the scientific conclusions of the article in any way. The original article has been updated.

