# Supplementary material for: Prognostic Ferroptosis-Related lncRNA Signatures Associated With Immunotherapy and Chemotherapy Responses in Patients With Stomach Cancer
Source: Front Genet. 2022 Jan 3;12:798612. doi: 10.3389/fgene.2021.798612 (PMC8762254; doi:10.3389/fgene.2021.798612)
Supplement: Supplementary file 1 [file DataSheet1.zip › Table S2.docx]

**Table S2|** 10 lncRNAs were screened in the MultiCox analysis.

| **lncRNAs** | **coef** | **HR** | **Lower 95% CI** | **Upper 95% CI** | **P.Value** |
| --- | --- | --- | --- | --- | --- |
| AC009299.2 | 2.214345 | 9.155407 | 1.561439 | 53.6822 | 0.01414 |
| AC012020.1 | -0.8597 | 0.42329 | 0.198248 | 0.903785 | 0.02633 |
| AC092723.2 | 0.683934 | 1.981658 | 1.296819 | 3.028155 | 0.00157 |
| AC093642.1 | 1.037749 | 2.822854 | 1.392715 | 5.721565 | 0.00399 |
| AC243829.4 | -1.18141 | 0.306845 | 0.135273 | 0.69603 | 0.0047 |
| AL121748.1 | 0.815962 | 2.261351 | 1.020605 | 5.010467 | 0.04441 |
| FLNB-AS1 | 0.188412 | 1.207331 | 1.046358 | 1.393068 | 0.00986 |
| LINC01614 | 0.058078 | 1.059798 | 1.017206 | 1.104173 | 0.00552 |
| LINC02485 | 0.384846 | 1.469388 | 1.06478 | 2.027742 | 0.01918 |
| LINC02728 | 0.441505 | 1.555047 | 1.236273 | 1.956017 | 0.00016 |
