# Supplementary figures and images for: Prognostic Ferroptosis-Related lncRNA Signatures Associated With Immunotherapy and Chemotherapy Responses in Patients With Stomach Cancer
Source: Front Genet. 2022 Jan 3;12:798612. doi: 10.3389/fgene.2021.798612 (PMC8762254; doi:10.3389/fgene.2021.798612)

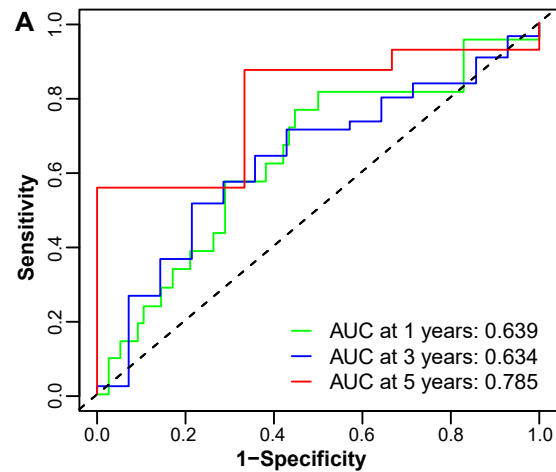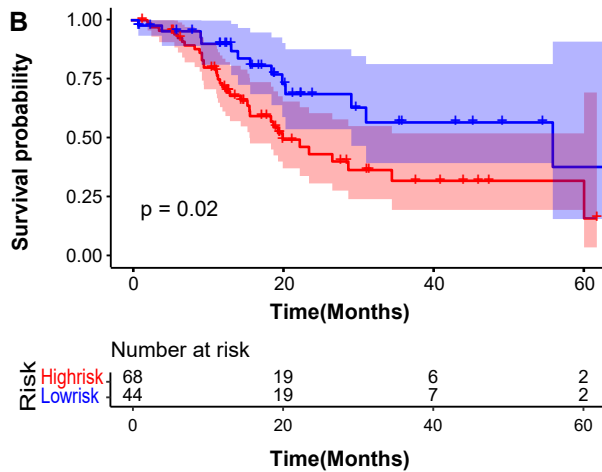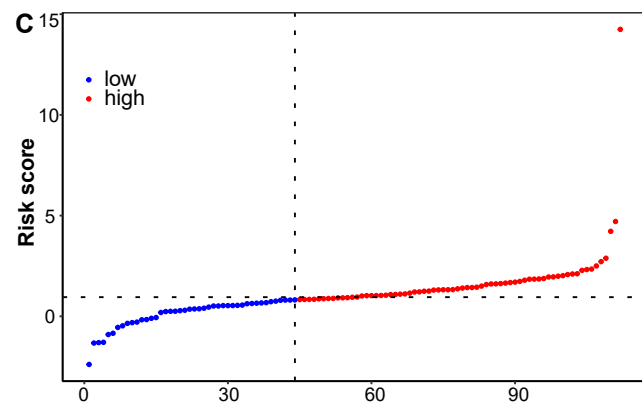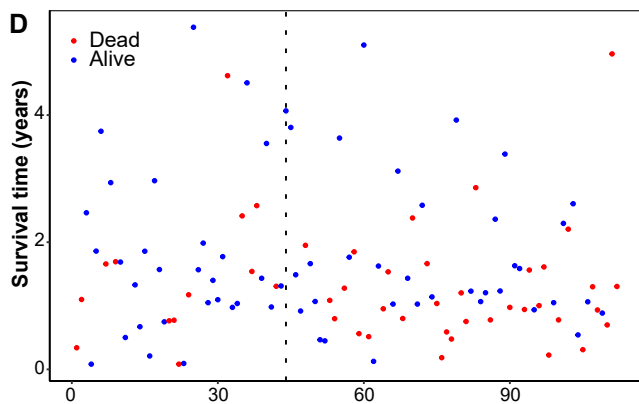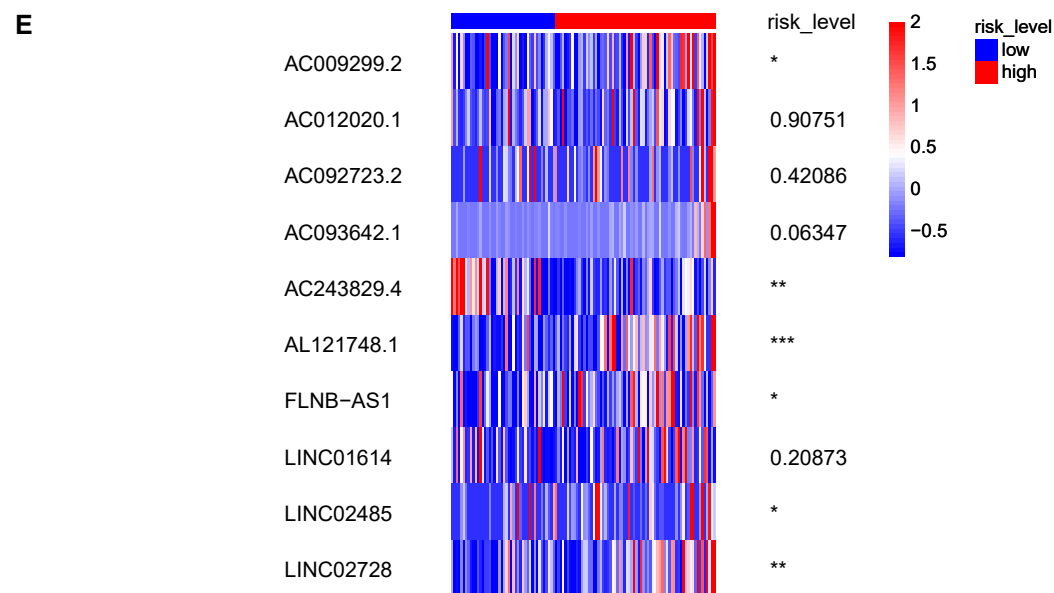

Supplement: Supplementary file 1 [file DataSheet1.zip › FigureS1.pdf]

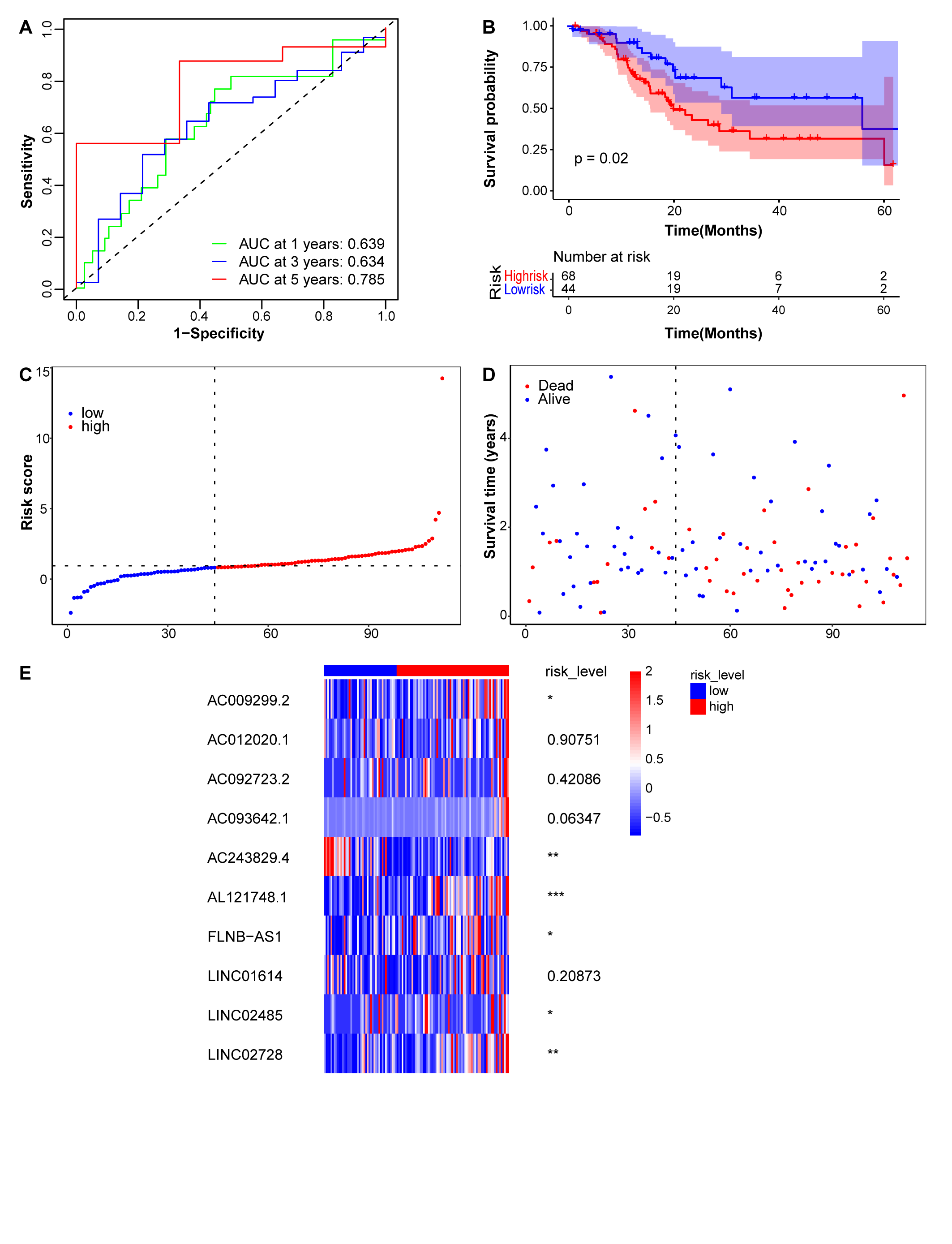

Supplement: Supplementary file 1 [file DataSheet1.zip › FigureS1.tif]

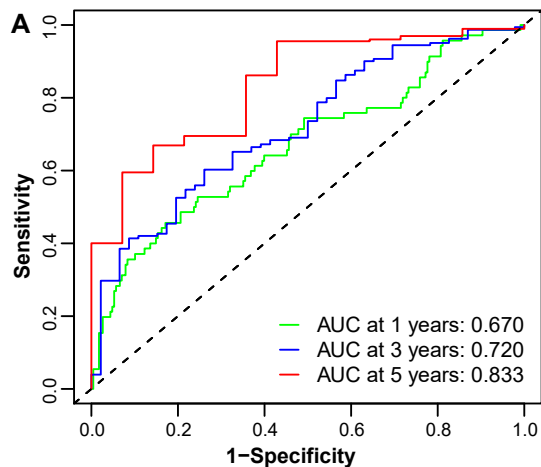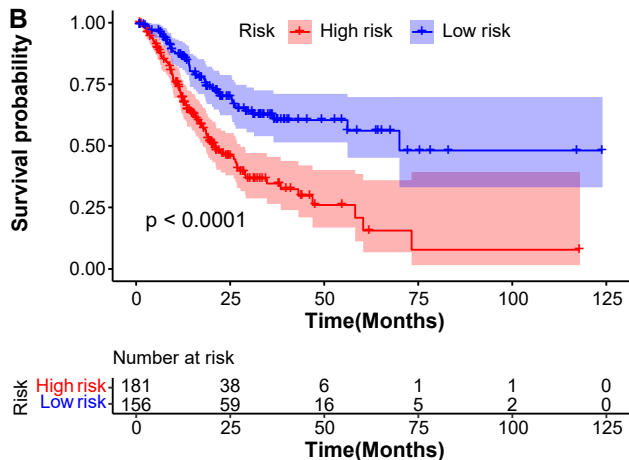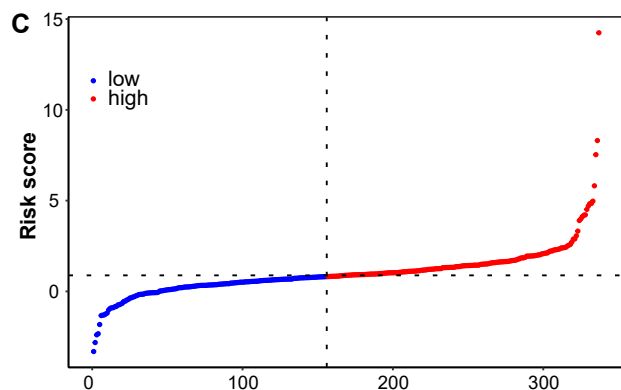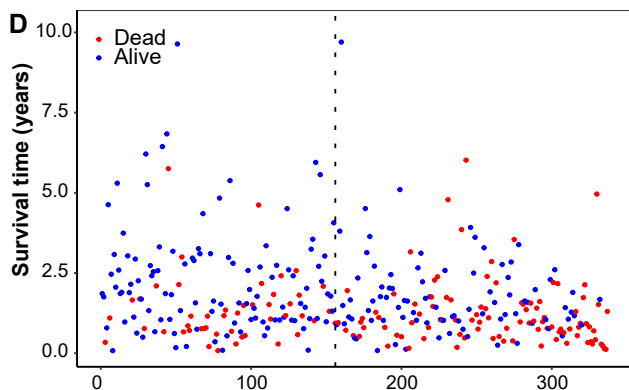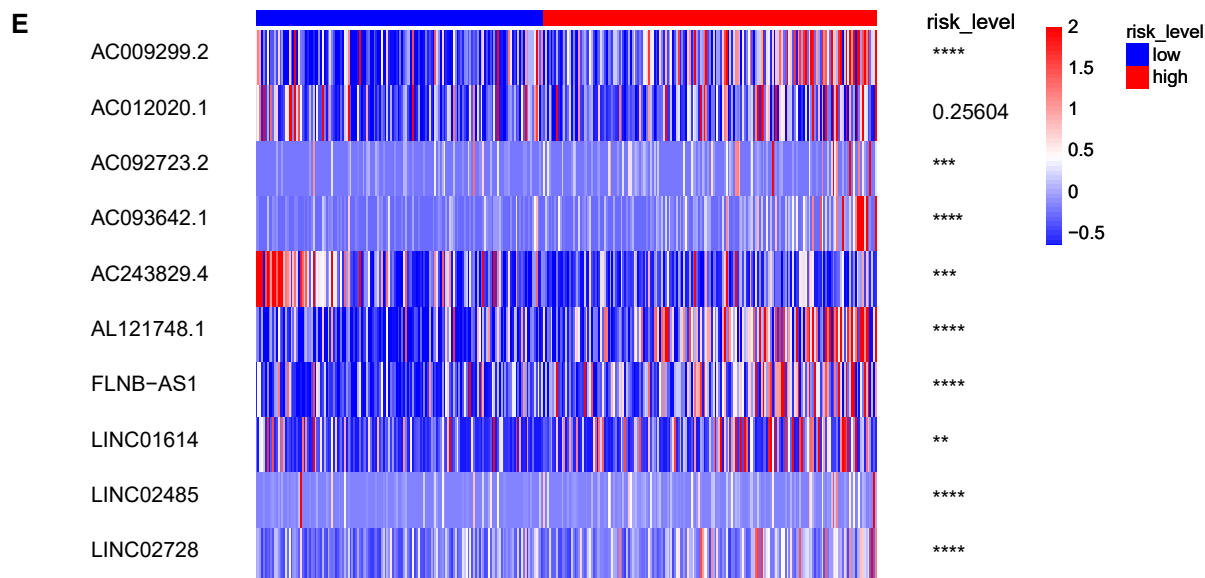

Supplement: Supplementary file 1 [file DataSheet1.zip › FigureS2.pdf]

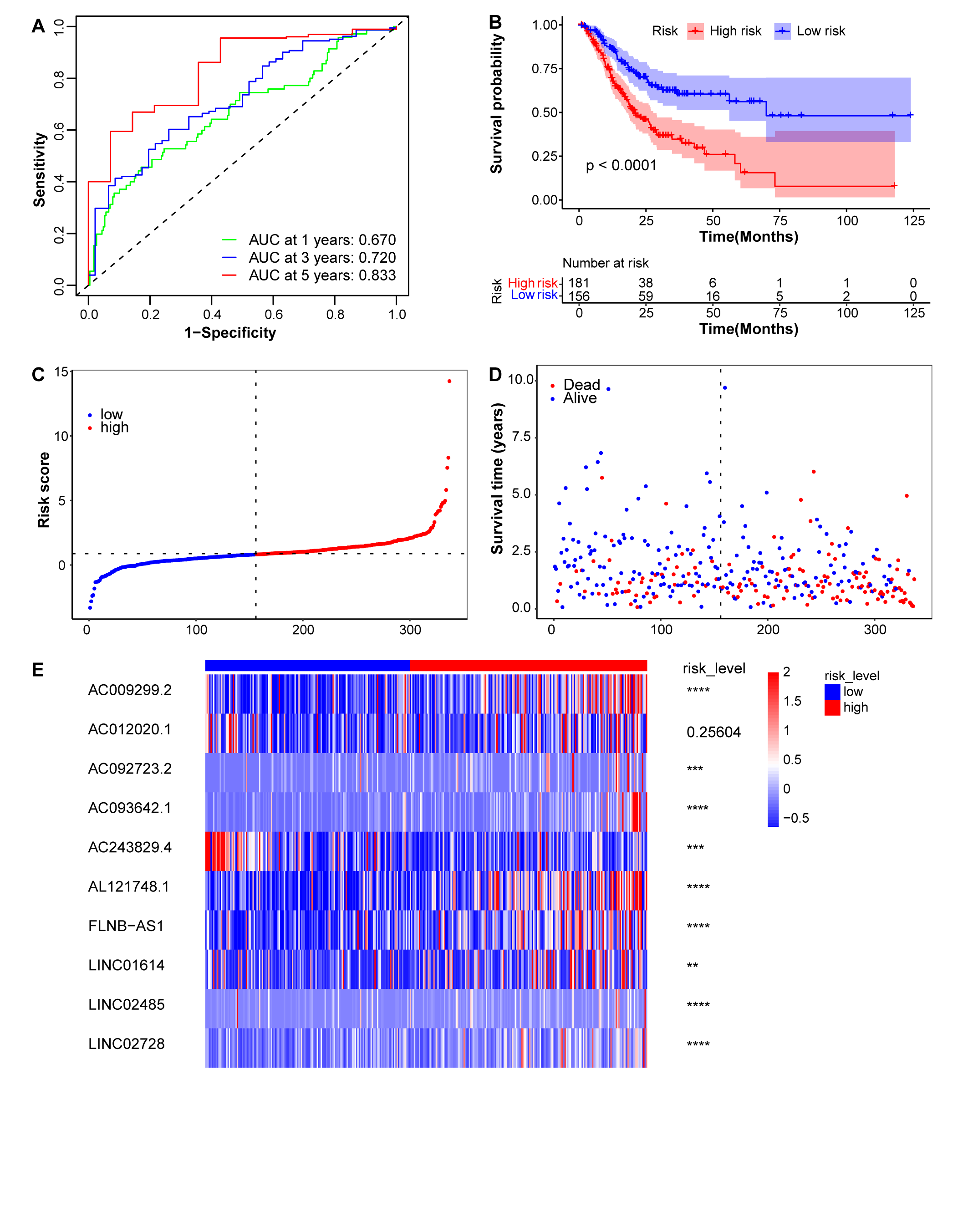

Supplement: Supplementary file 1 [file DataSheet1.zip › FigureS2.tif]

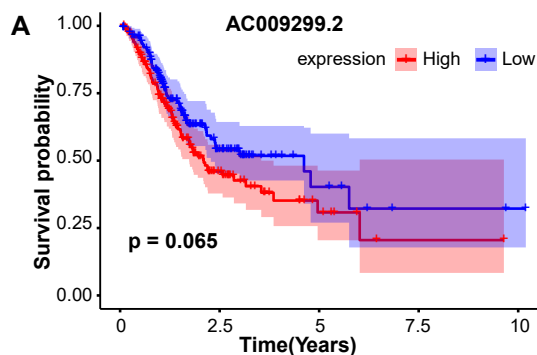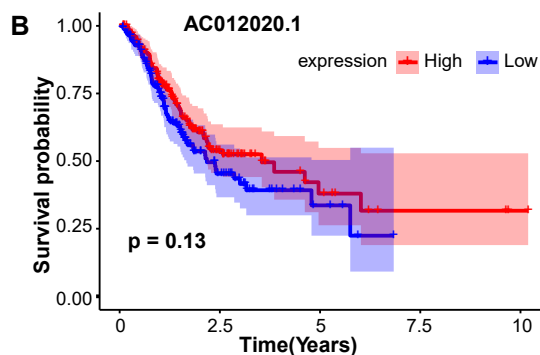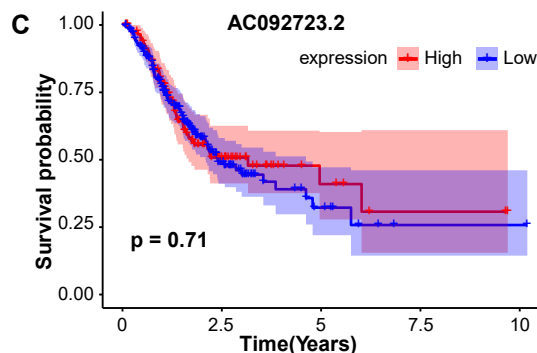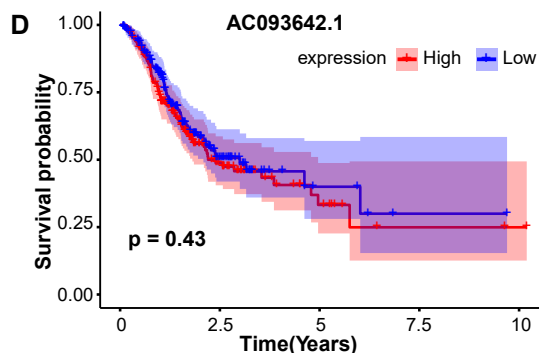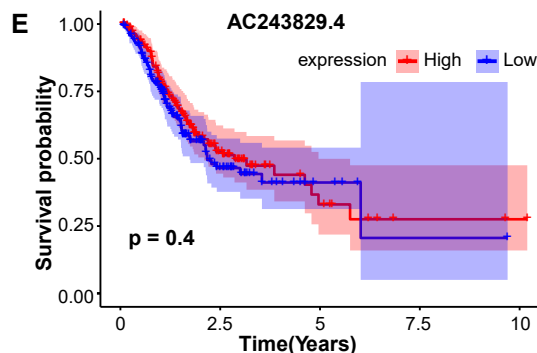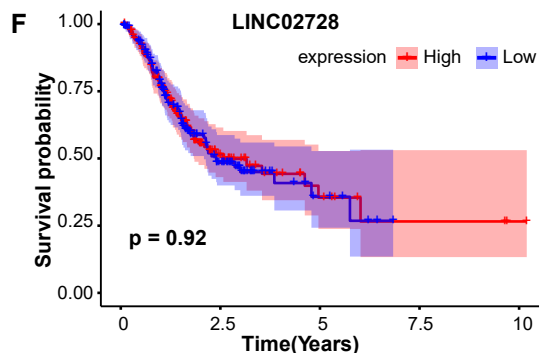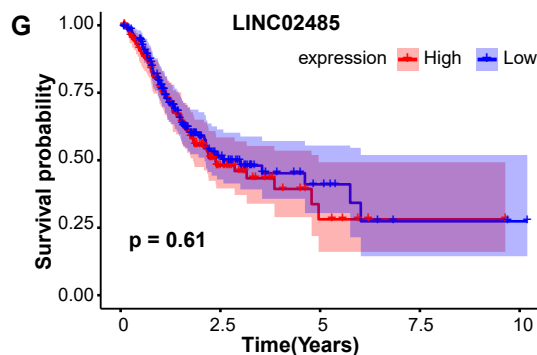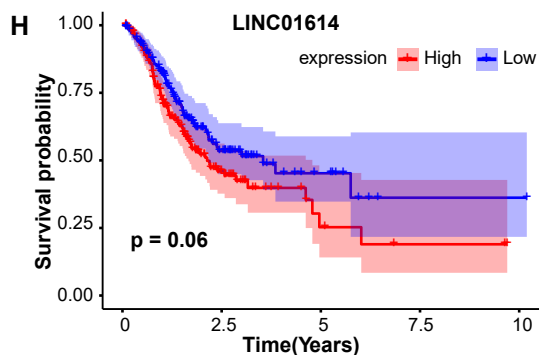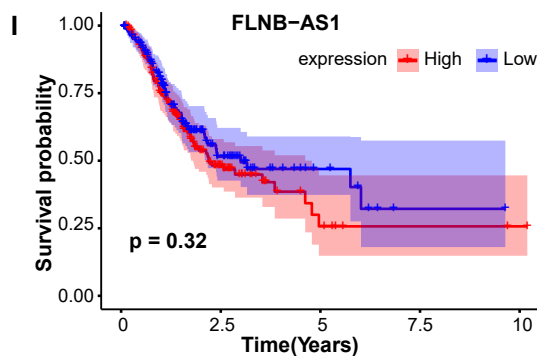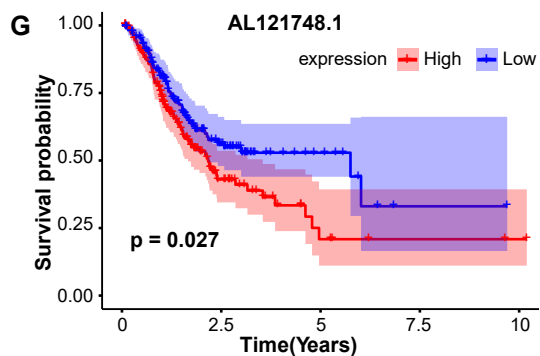

Supplement: Supplementary file 1 [file DataSheet1.zip › FigureS3.pdf]

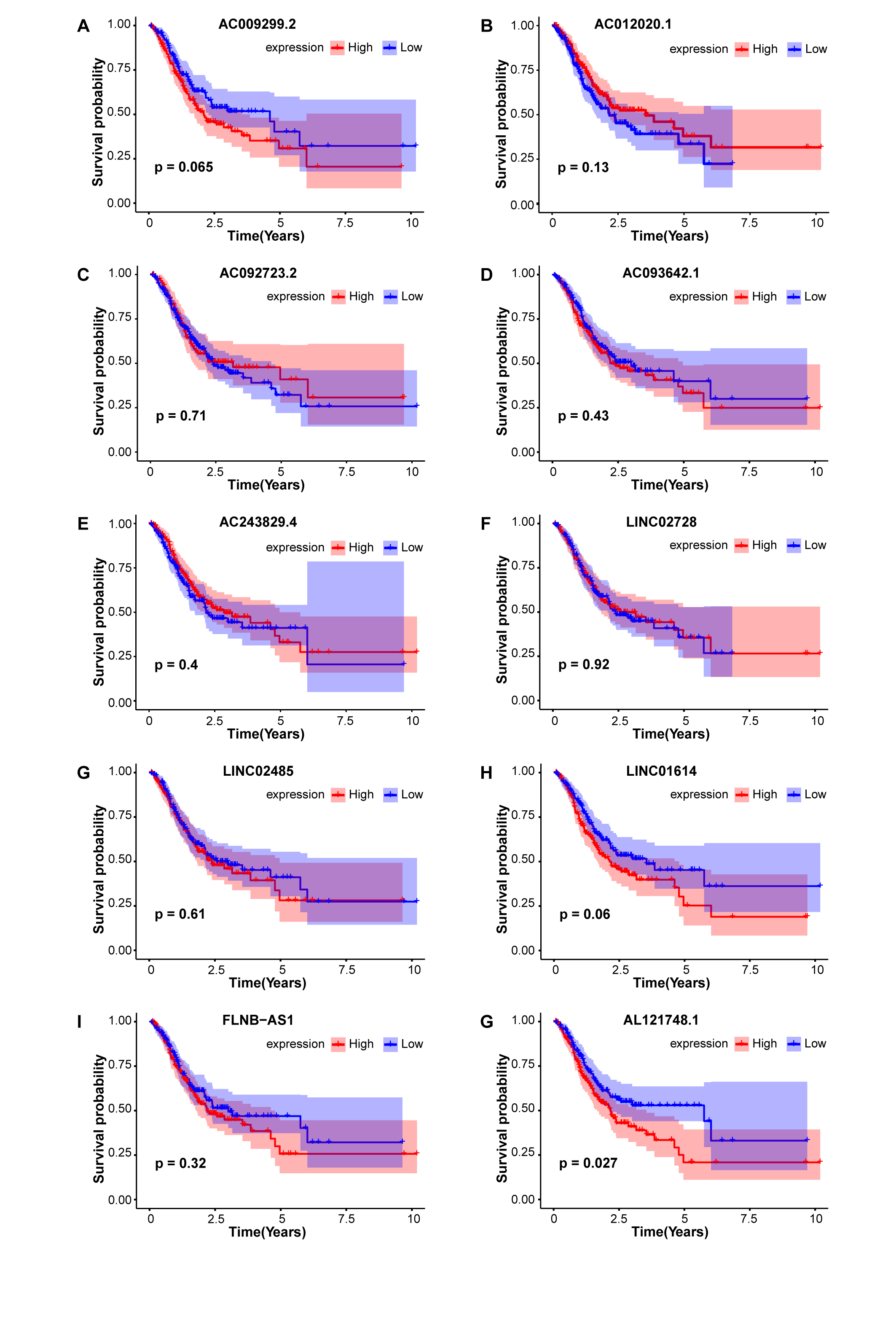

Supplement: Supplementary file 1 [file DataSheet1.zip › FigureS3.tif]

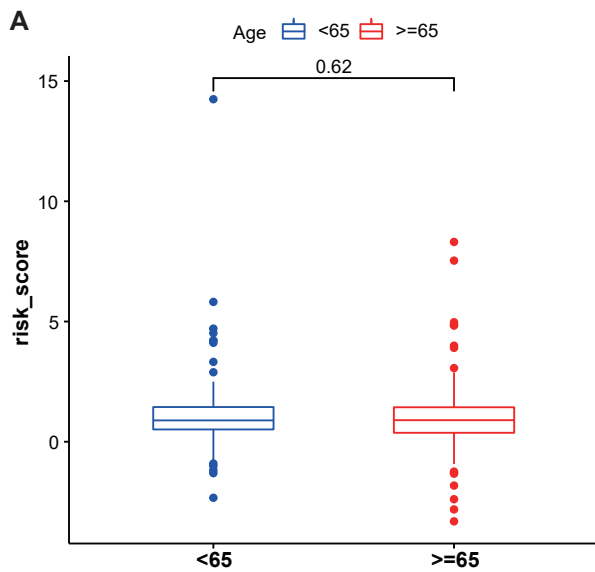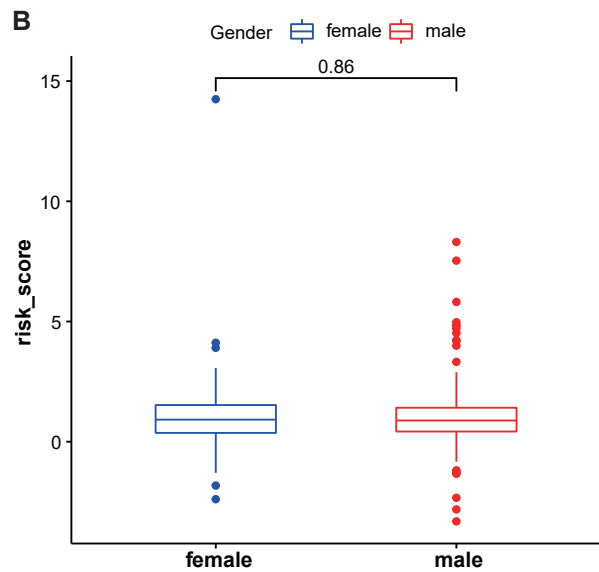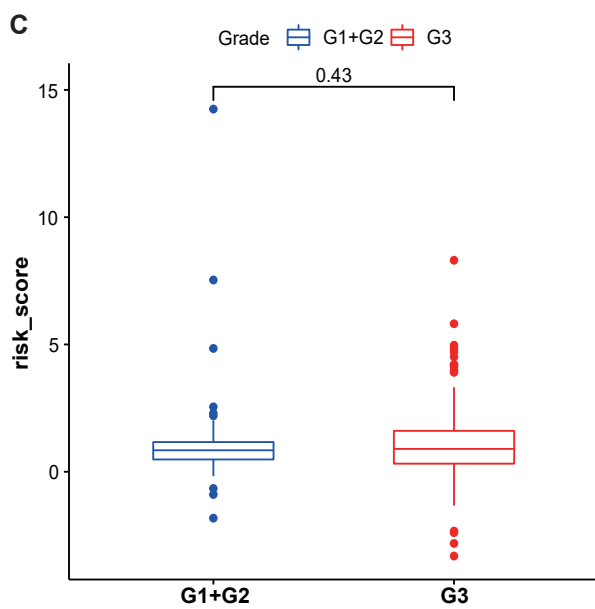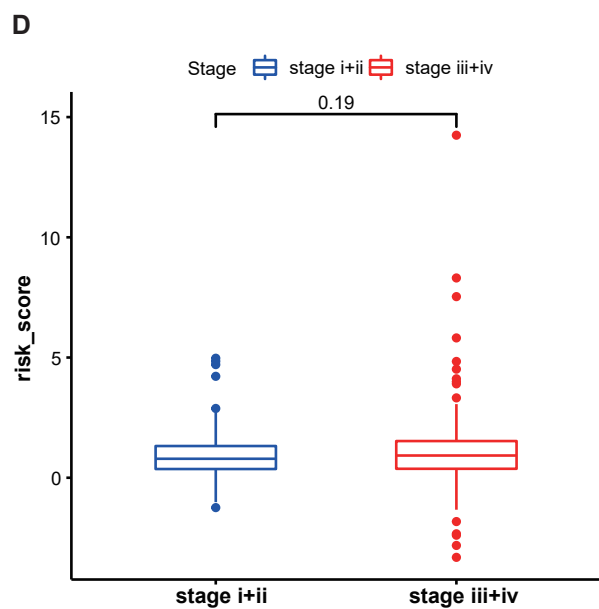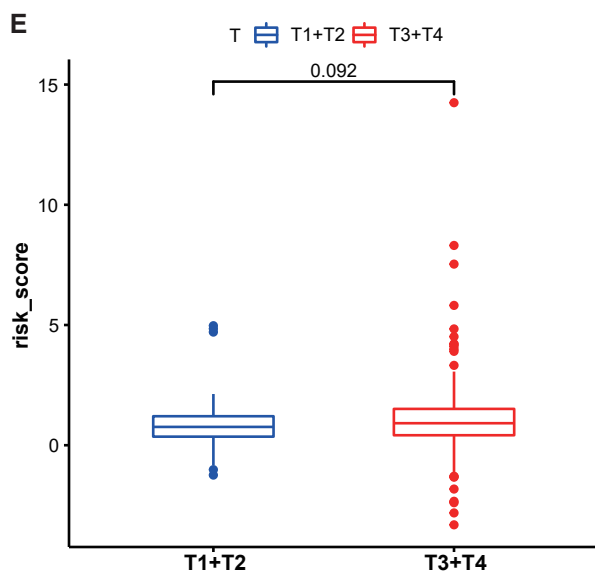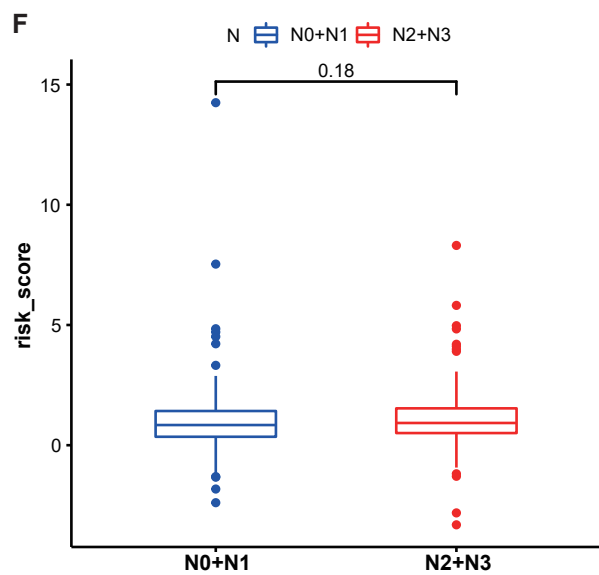

Supplement: Supplementary file 1 [file DataSheet1.zip › FigureS4.pdf]

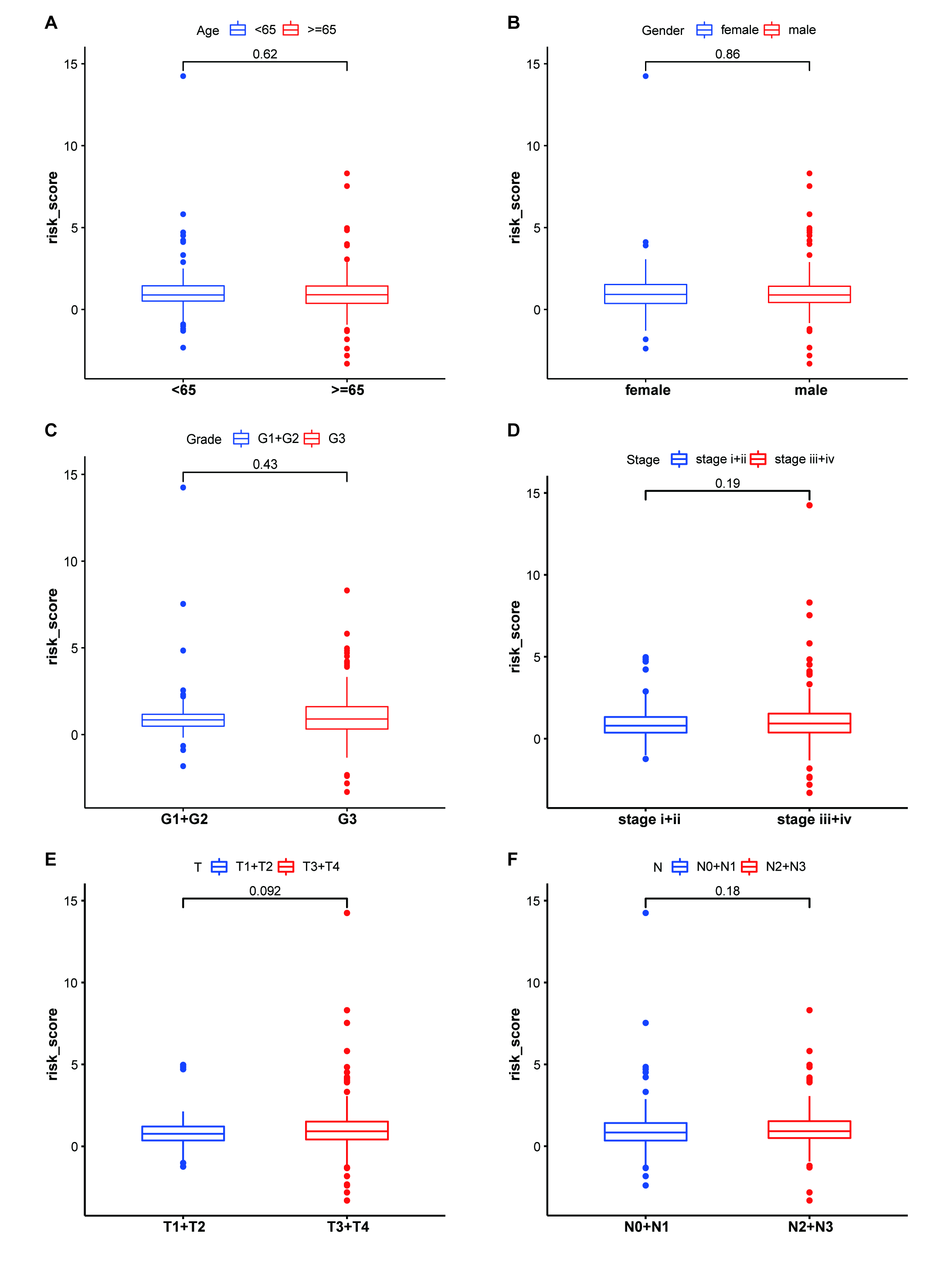

Supplement: Supplementary file 1 [file DataSheet1.zip › FigureS4.tif]

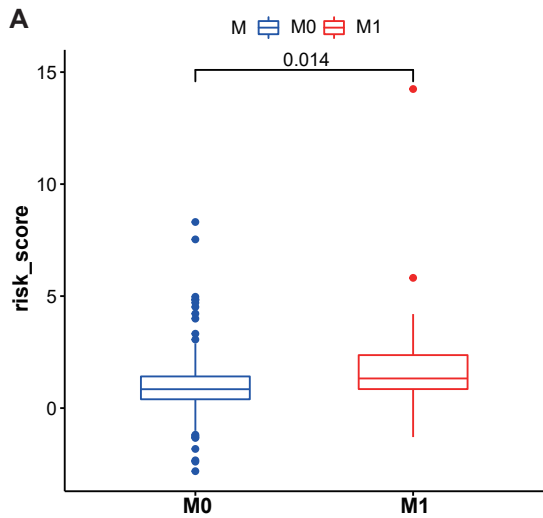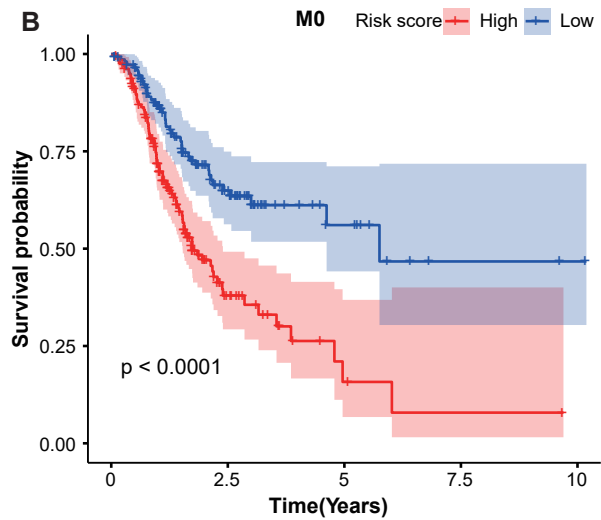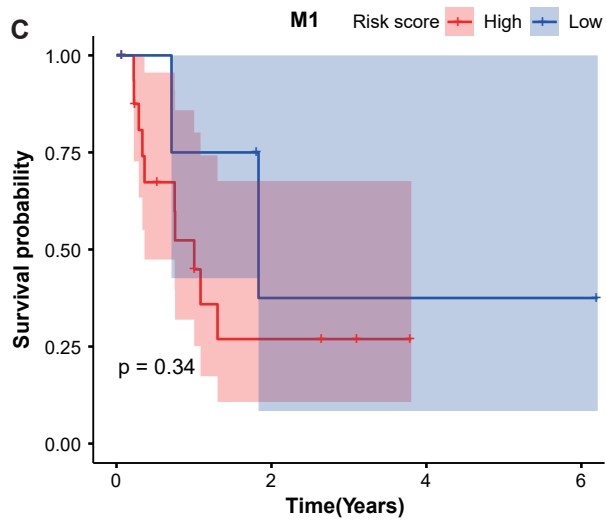

Supplement: Supplementary file 1 [file DataSheet1.zip › FigureS5.pdf]

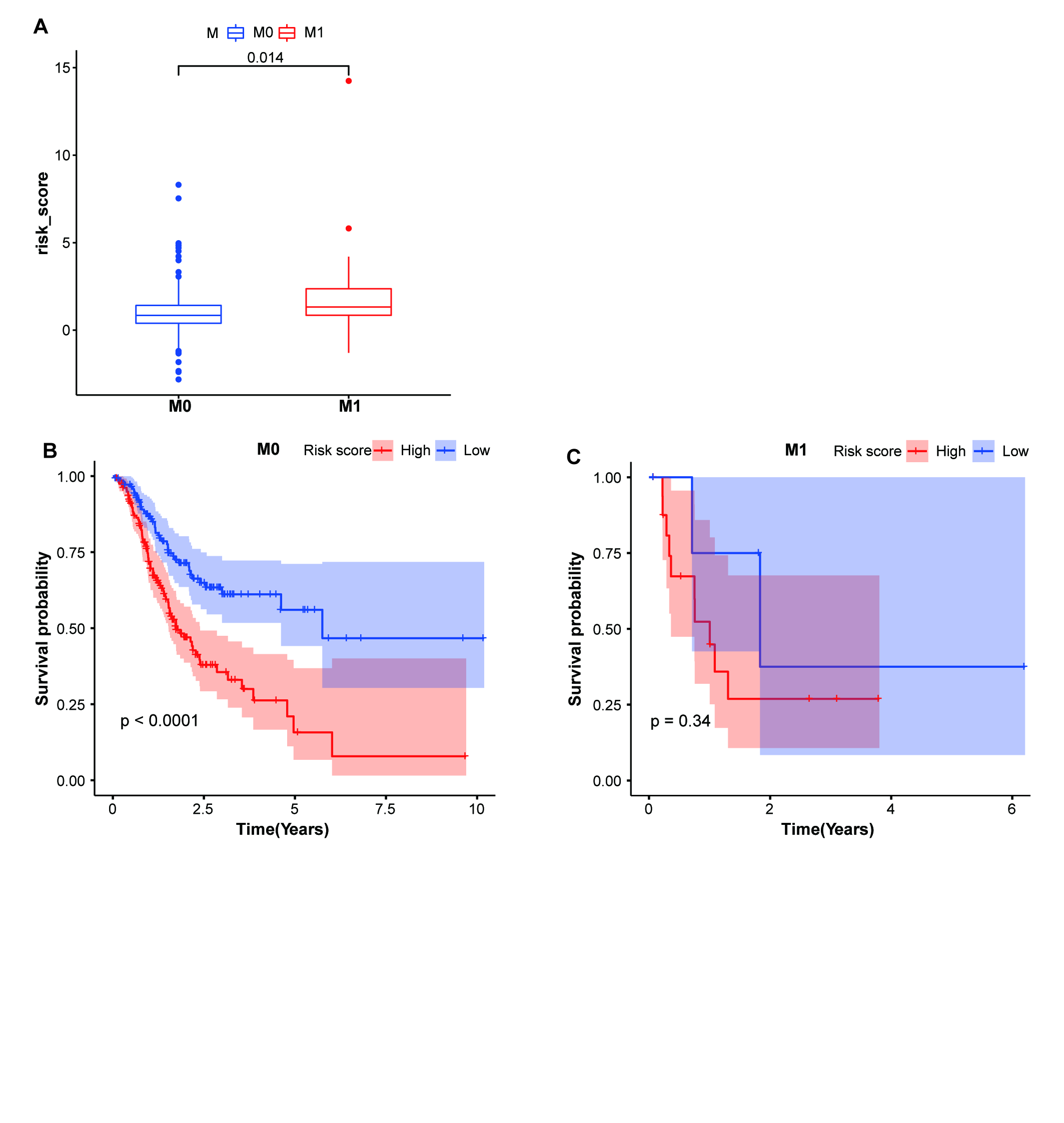

Supplement: Supplementary file 1 [file DataSheet1.zip › FigureS5.tif]

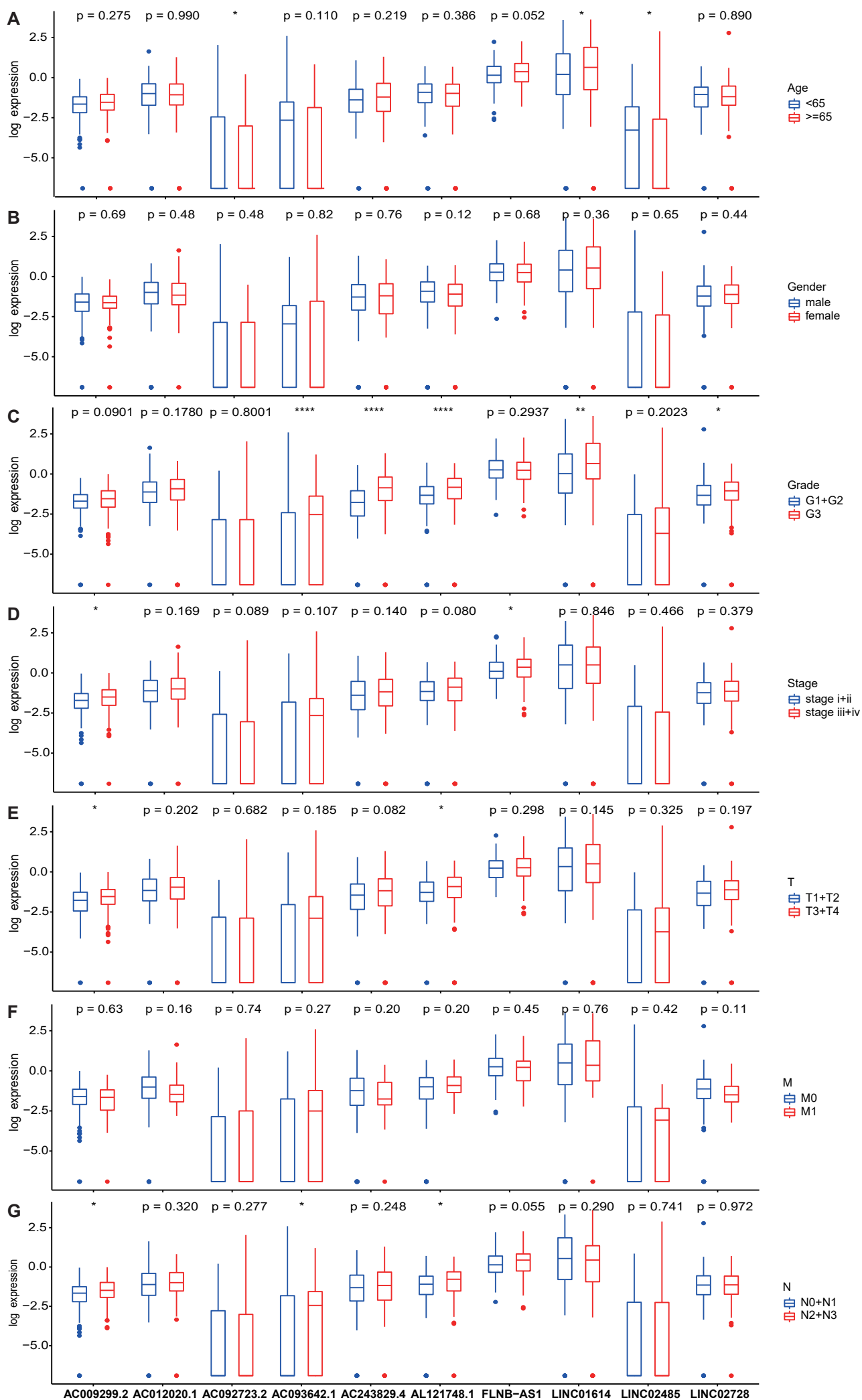

Supplement: Supplementary file 1 [file DataSheet1.zip › FigureS6.pdf]

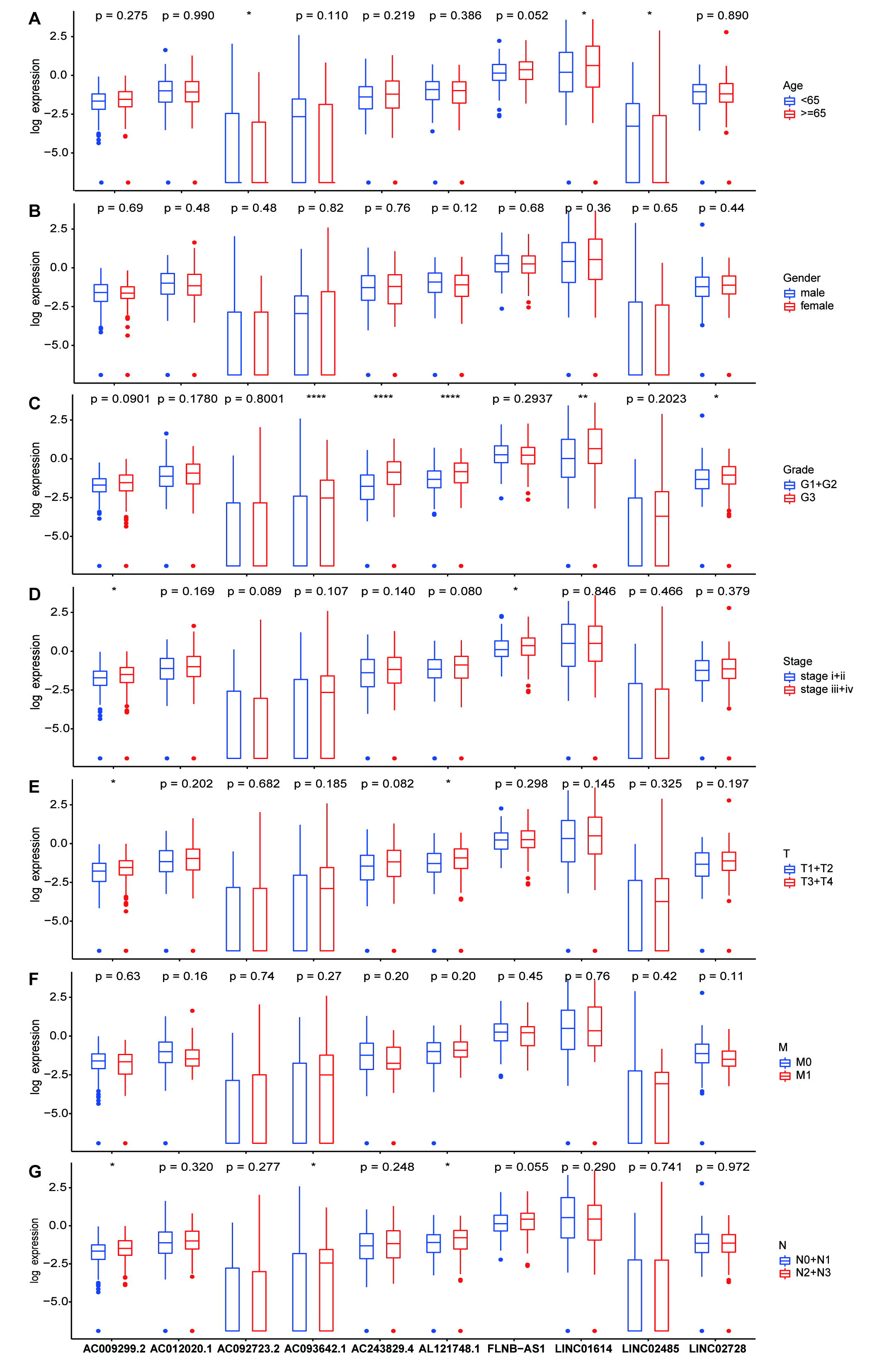

Supplement: Supplementary file 1 [file DataSheet1.zip › FigureS6.tif]

**A**

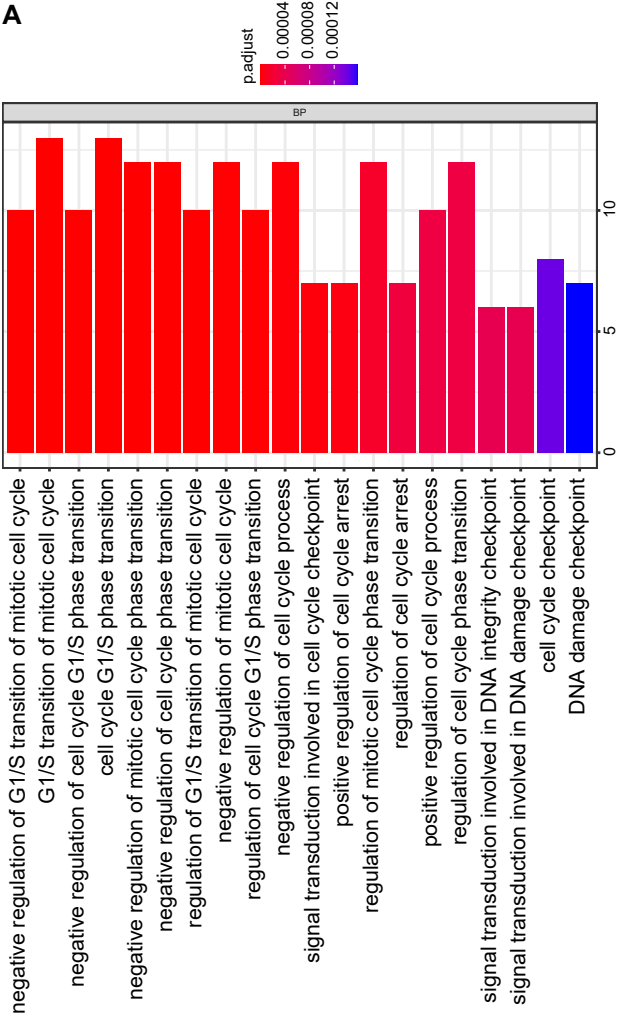

**B**

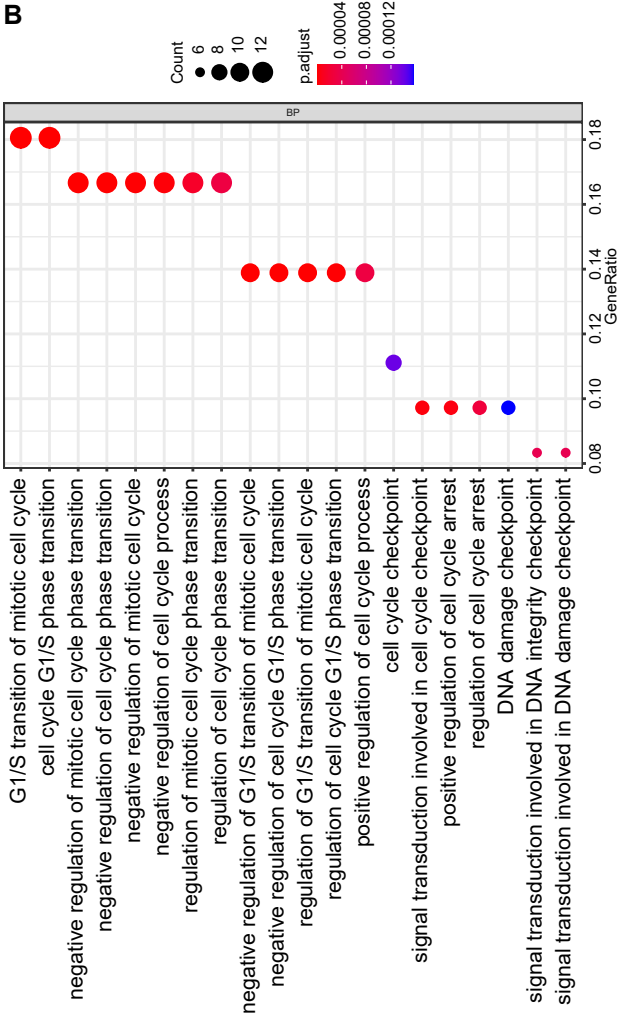

**C**

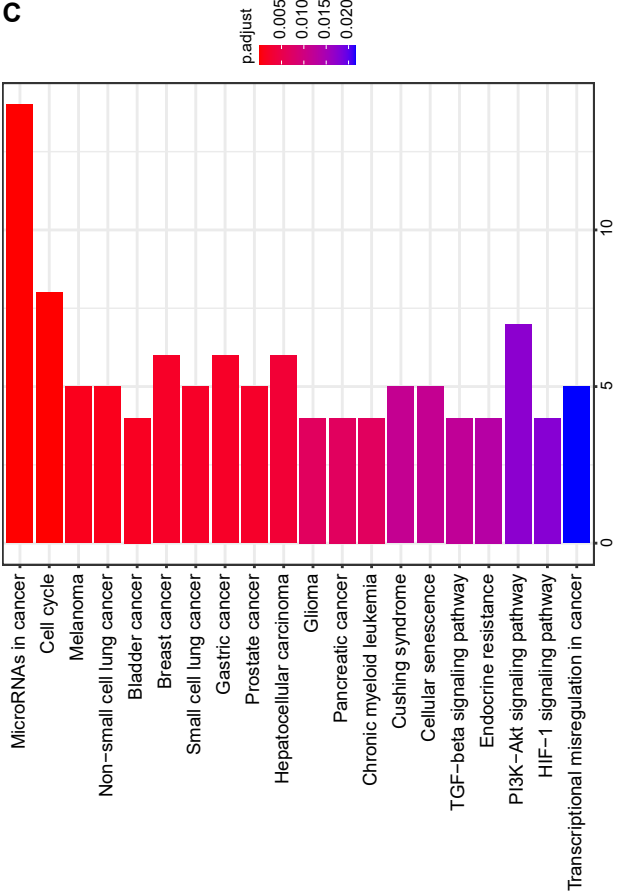

**D**

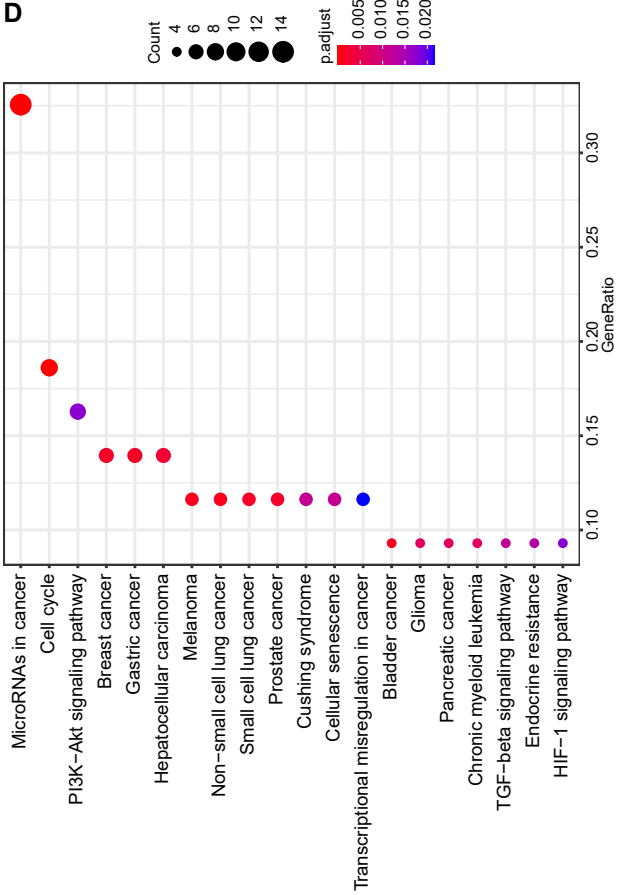

Supplement: Supplementary file 1 [file DataSheet1.zip › FigureS7.pdf]

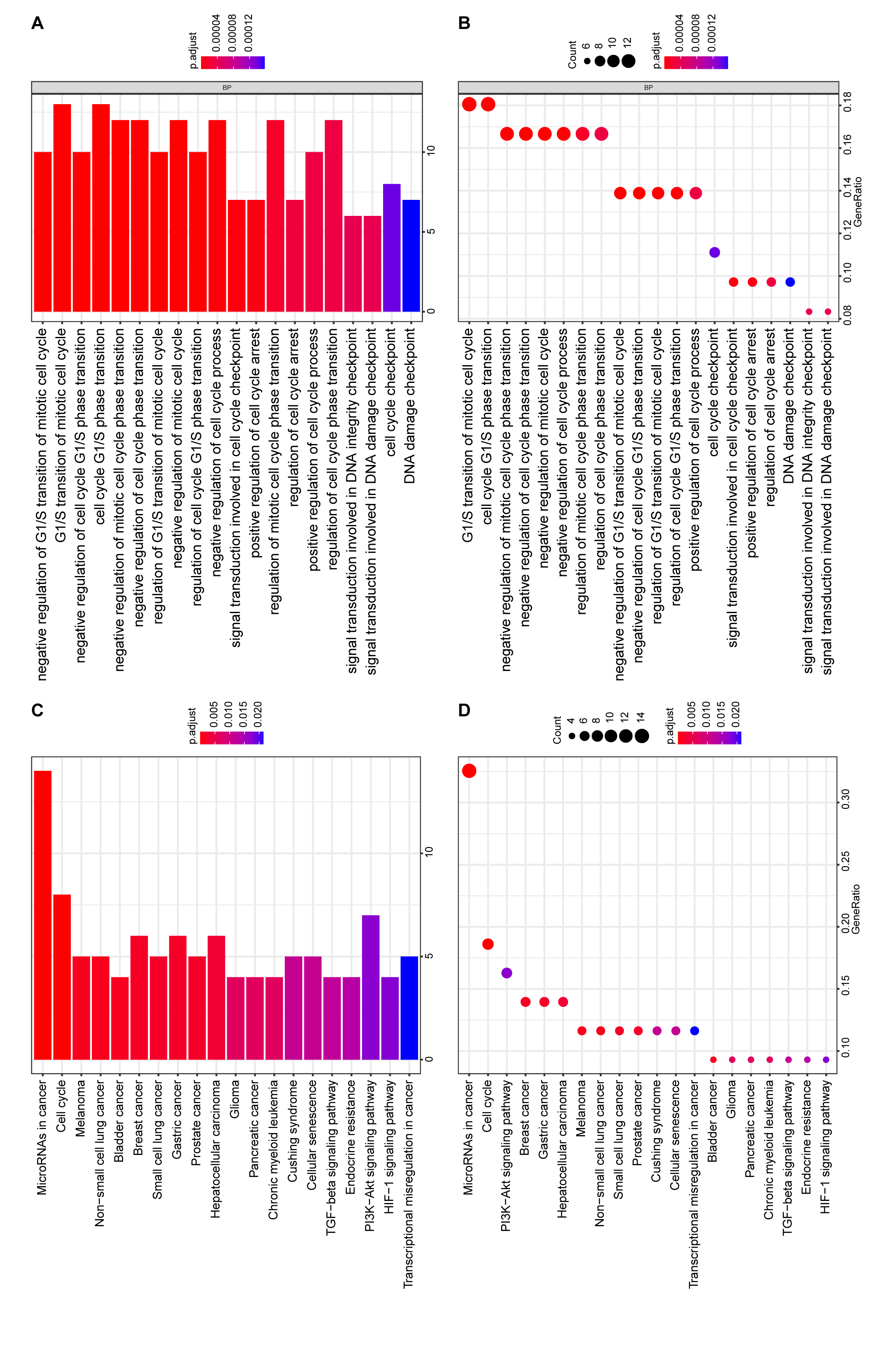

Supplement: Supplementary file 1 [file DataSheet1.zip › FigureS7.tif]

Altered in 290 (88.15%) of 329 samples.

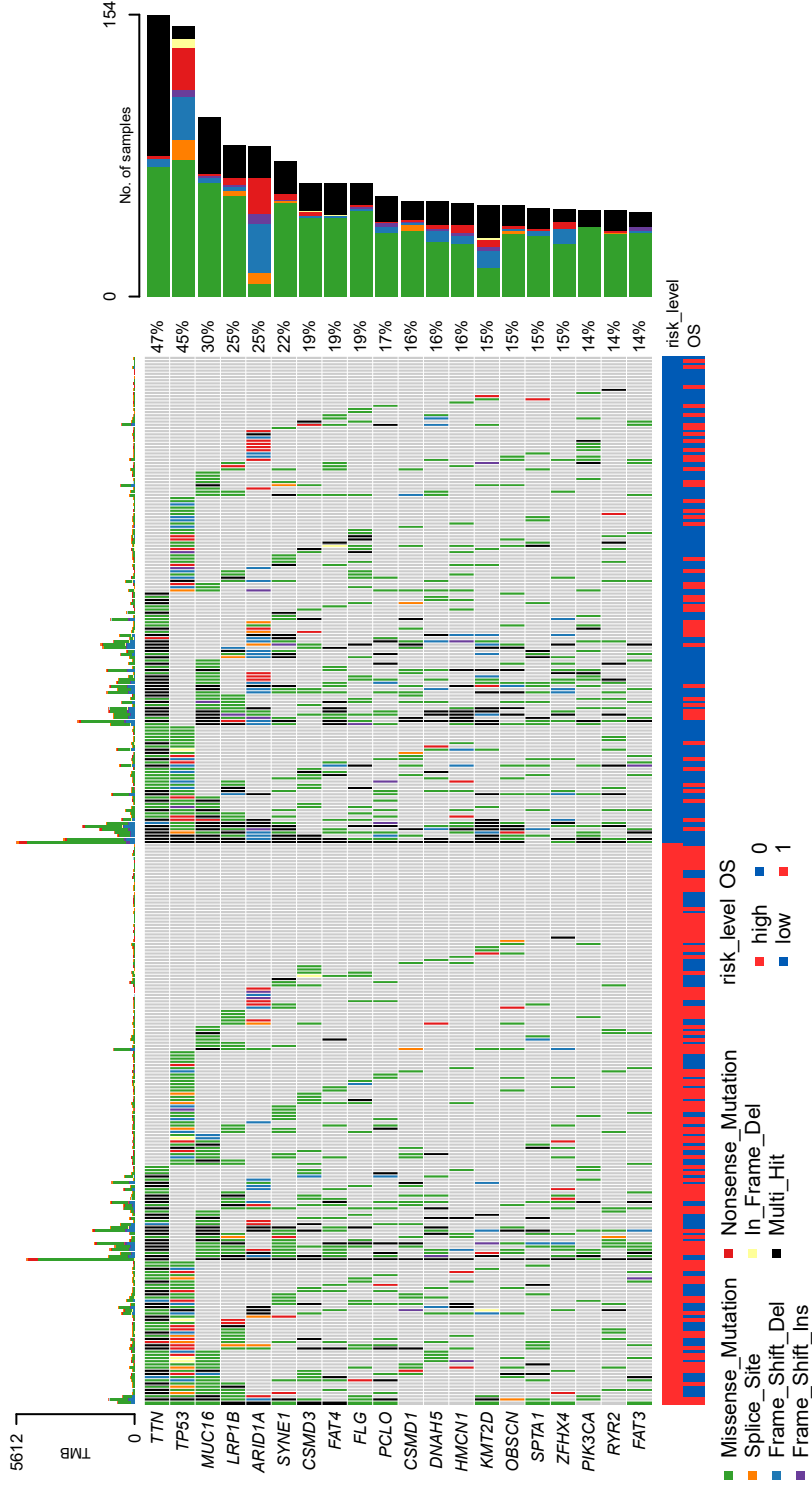

Supplement: Supplementary file 1 [file DataSheet1.zip › FigureS8.pdf]

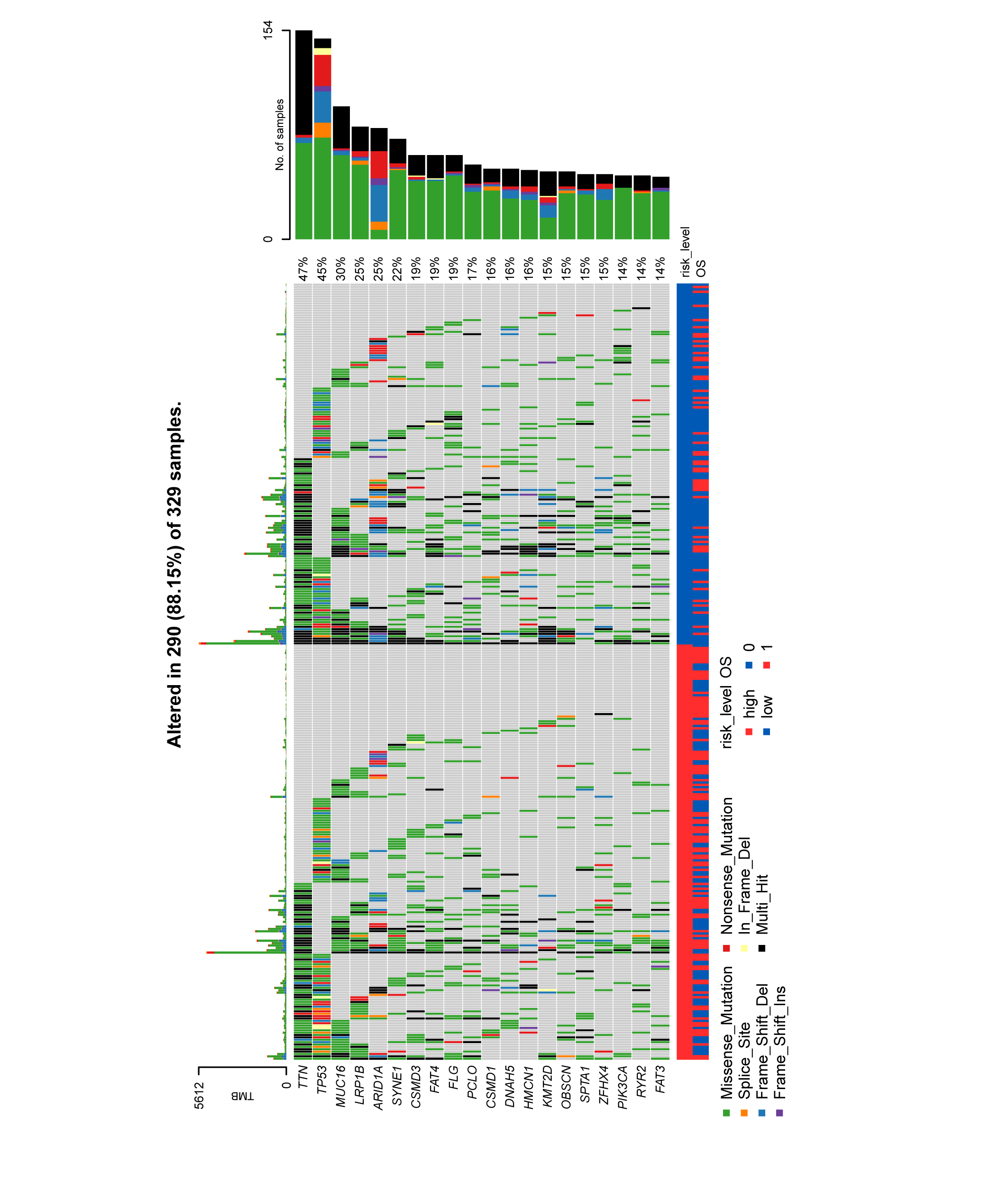

Supplement: Supplementary file 1 [file DataSheet1.zip › FigureS8.tif]
